# Fabrication of Polymeric Microparticles by Electrospray: The Impact of Experimental Parameters

**DOI:** 10.3390/jfb11010004

**Published:** 2020-01-15

**Authors:** Alan Í. S. Morais, Ewerton G. Vieira, Samson Afewerki, Ricardo B. Sousa, Luzia M. C. Honorio, Anallyne N. C. O. Cambrussi, Jailson A. Santos, Roosevelt D. S. Bezerra, Josy A. O. Furtini, Edson C. Silva-Filho, Thomas J. Webster, Anderson O. Lobo

**Affiliations:** 1 LIMAV—Interdisciplinary Advanced Materials Laboratory, PPGCM—Materials Science and Engineering Graduate Program, UFPI—Federal University of Piaui, Teresina 64049-550, Brazil; alanicaro@gmail.com (A.Í.S.M.); ewertonvieira@ufpi.edu.br (E.G.V.); luzia_quimica@yahoo.com.br (L.M.C.H.); anallynecambrussi@gmail.com (A.N.C.O.C.); j.santospi@hotmail.com (J.A.S.); josy_osajima@yahoo.com.br (J.A.O.F.); edsonfilho@ufpi.edu.br (E.C.S.-F.); 2 Division of Engineering in Medicine, Department of Medicine, Harvard Medical School, Brigham & Women’s Hospital, Cambridge, MA 02139, USA; samsom.afewerki@gmail.com; 3 Harvard-MIT Division of Health Science and Technology, Massachusetts Institute of Technology, MIT, Cambridge, MA 02139, USA; 4 Federal Institute of Education, Science and Technology of Tocantins, Dianápolis Campus, IFTO, Dianápolis 77300-000, Tocantins, Brazil; ricardo.sousa@ifto.edu.br; 5 Federal Institute of Education, Science and Technology of Piauí, Teresina-Central Campus, IFPI, Teresina 64000-040, Brazil; rooseveltdsb@ifpi.edu.br; 6 Department of Chemical Engineering, Northeastern University, Boston, MA 02115, USA; th.webster@northeastern.edu

**Keywords:** microparticles, polymers, electrospray, operational parameters, biomaterials

## Abstract

Microparticles (MPs) with controlled morphologies and sizes have been investigated by several researchers due to their importance in pharmaceutical, ceramic, cosmetic, and food industries to just name a few. In particular, the electrospray (ES) technique has been shown to be a viable alternative for the development of single particles with different dimensions, multiple layers, and varied morphologies. In order to adjust these properties, it is necessary to optimize different experimental parameters, such as polymer solvent, voltage, flow rate (FR), type of collectors, and distance between the collector and needle tip, which will all be highlighted in this review. Moreover, the influence and contributions of each of these parameters on the design and fabrication of polymeric MPs are described. In addition, the most common configurations of ES systems for this purpose are discussed, for instance, the main configuration of an ES system with monoaxial, coaxial, triaxial, and multi-capillary delivery. Finally, the main types of collectors employed, types of synthesized MPs and their applications specifically in the pharmaceutical and biomedical fields will be emphasized. To date, ES is a promising and versatile technology with numerous excellent applications in the pharmaceutical and biomaterials field and such MPs generated should be employed for the improved treatment of cancer, healing of bone, and other persistent medical problems.

## 1. Introduction

In recent decades, interest in organic microparticles (MPs) has grown rapidly due to their important applications in various industries such as the pharmaceutical, ceramic, cosmetic, and food industries [1,2]. Between these and various other industrial fields of interest, one can take advantage of MP production in a controlled manner, therefore, the production of these materials has been attempted through several approaches [1,3]. For instance, jet-milling [4], spray drying [5], micro grinding [6], suspension/emulsion polymerization [7], solvent evaporation [8], microfluidics [9], and electrospray (ES) have all been studied for optimal MP fabrication [10]. Hence, typically, such MPs are manufactured using polymers as the desirable properties of selected polymers (i.e., mechanical, diffusion, electrical, biodegrability, etc.) may be beneficial in adapting particle functionality for such specific applications [1]. 

One of the main applications of MPs is in the area of healthcare, such as for the engineering of drug- or protein-loaded MPs. However, there are some limitations of the above-mentioned MP fabrication techniques, as in the case of “double emulsion methods” which consists of different interfaces such as the water/oil/water (W/O/W) method, the solid/oil/water (S/O/W) method, the water/oil/oil (W/O/O) method (coacervation method), and the solid/oil/oil (S/O/O) method [11]. For instance, the main limitation with the W/O/W method is solvent removal which is a critical step which reduces loading capacity and encapsulation efficiency. Another example is spray drying in which high operating temperatures, separation of synthesized particles and loss of products using a commercial scale laboratory spray dryer are some significant limitations and challenges [11]. Among these methods, ES technology has attracted a great deal of attention as it is an easy and suitable method for the formation of polymer particles with highly controllable sizes, dispersions, unconventional shapes and unique surface morphologies that cannot be achieved by other conventional MP fabrication methods [1]. In this context, Table 1 demonstrates some advantages and disadvantages between the aforementioned techniques and ES technology.

ES is a facile, simple and direct approach, and therefore, a promising technology for the engineering of MPs suitable for many translational applications. In addition, it is of significant importance for the biomedical field since it allows for the facile encapsulation of drugs in MPs [12,13], and the controlled processing of biodegradable polymeric MP [14]. In this review, we aim to discuss and highlight various parameters employed in ES technology for the manufacturing of polymeric MPs; their impact on MP properties; the influence of processing parameters such as polymer solvent, applied voltage, flow rate (FR), types of collectors, distance between the collector and the tip of the needles, etc.; the types of MPs produced through this technique; and its applications particularly in medicine. For example, in the work of Wang et al., it was shown that the concentration of the polymeric solution has great influence on the formation of MPs, in which a polymer (poly(methyl methacrylate) (PMMA)) and solvent (*N*,*N*-dimethylformamide (DMF)) were used [15]. Another example, in a study performed by Yao et al. [16], demonstrated that a low FR of the polymer solution led to the formation of dry particles but on the other hand, a high FR resulted in wet particles. Consequently, showing that these among other processing parameters have great influence on the formation of the final material, in this case the MPs, with different sizes and morphologies emphasizes a heightened attention that needs to be paid to ES systems. We hope that these fundamental questions will boost the understanding, tailorability and application of the ES technology to numerous fields. To conclude, this review focuses on using ES for the production of MPs and associated experimental parameters that impact the engineering and application of MPs.

## 2. Electrospray (ES)

**Figure 1:**
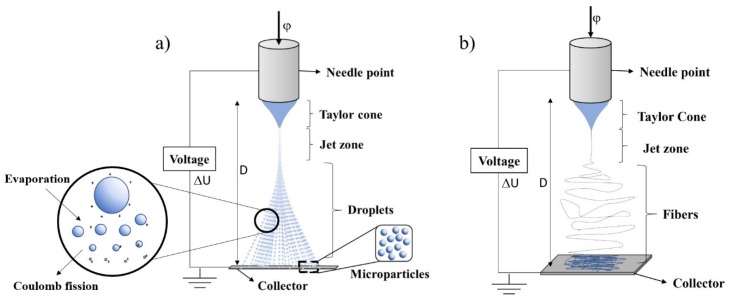
(**a**) Demonstration of the electrospray (ES) process, where a stable Taylor cone is formed from the needle tip injection. (**b**) Demonstration of Electrospinning (ESP) technology, where a stable Taylor cone can be observed. Flow rate (φ), potential difference (ΔU), and working distance (D).

Electrohydrodynamic atomization (EHDA) is an interdisciplinary field and a subfield of fluid mechanics dealing with the effects of applied electrical forces on liquids [17,18]. Processes proceed by applying an electrical charge to a viscous liquid through a capillary nozzle, leading to the formation of droplets and further atomizing particles to possess varying sizes from hundreds of micrometers (μm) to several tens of nanometers (nm). This process which mainly contains an emitter and an electrode sustained at different electrical potential provides almost a monodispersed distribution of particles. ES is considered a part of EHDA [17,19]. Generally, ES consists of components such as a high voltage source, a syringe pump (SP), a spray head, and a collector [12,20,21]. ES proceeds by forcing a liquid through a capillary to a collector under a potential drop, on the order of kilovolts (kV), which is applied between the capillary and the collector [22]. Typically, a liquid of non-zero electrical conductivity is expelled through a low flow capillary (mL/h) and a high voltage difference (kV). As depicted in Figure 1, this is established between the capillary and a counter electrode arranged at a certain distance from the capillary in the manifold. A high voltage applied to the needle is passed into a polymer solution which is then ejected from the nozzle. When the polymer solution flows through the capillary, the droplet is highly electrified and distorted into a conical shape commonly known as a Taylor cone (Figure 1). After the solvents are evaporated during the course of the jet spray, the generated electrospun polymeric materials are collected on or in a grounded collector [21]. Here, in the ES technique, the main aspect consists on the deformation of the liquid flow at the emitter output, in the case of the needle tip, acquiring a conical structure (Taylor cone), due to EHDA interactions, as shown in Figure 1 [17,23,24]. Figure 1 also demonstrates the difference between the ES and Electrospinning (ESP) techniques, where ES is a variant of the method of ESP, which began in medicine with an aim to easily and quickly encapsulate drugs (Figure 1b) [25]. The basic difference between these technologies is the concentration of the polymer used and the molecular weight, i.e., when high polymer concentrations are employed, there is a tendency for yarn to be formed (Figure 1b), and for lower polymer concentrations, particle formation can be observed [15].

In order to generate monodispersed droplets, ES needs to be maintained, ensuring the Taylor cone stays stable and then further forms a very fine jet emerging from the tip of the cone. Subsequently, the Taylor cone emits a jet of liquid leading to the separation and the generation of droplets, forming a cloud of tiny droplets charged with a narrow size distribution. The droplets are promoted by the electric field and evaporate along its path, leaving a solid residue of charged particles that are obtained in a collector [17,26]. The principles of ES can be grounded into the theory of charged drops, which in its concept, states that if an electric field is applied to a drop, the electric charge generates an electrostatic force on the drop (a Coulomb force), that competes with the cohesion force in the drop. Hence, when the Coulomb force overcomes the cohesive force of a drop, it results in the release of surface tension and particle formation [21,25]. Nevertheless, there is a limit, called the Rayleigh limit (L_R_), which exists to determine when the drop breaks. This limit is when the surface tension of the drop is exceeded by the electrostatic force. Equation (1) represents the Rayleigh limit [25]:

(1) $$\begin{array}{c} L_{r}=q\left(64\pi^{2}\varepsilon \gamma R^{3}\right)\\ q=8\pi \sqrt{\varepsilon_{0}\gamma R^{3}}) \end{array}$$

where *q* is the charge on the surface of the droplet, *ε* is the permittivity of the surrounding medium, γ is the surface tension of the fluid, and *R* is the radius of the droplet.

Therefore, it can be observed that in ES technology, there are several impactful factors present in the process that one needs to pay attention to, such as the viscosity of the liquid containing the polymeric solution, applied process voltage originating from the high voltage sources and collectors which collect the microparticles, among others. These are some examples of processing parameters which are altered to promote the formation of microparticles. Specifically, a complete list of ES processing parameters include settings such as types of needles, polymer concentration, solvent, solution conductivity, applied voltage, FR, electric field, temperature and humidity, collector distance (distance between the collector and the tip of the needles) and the type of collectors. Therefore, in the following sections, we will present and discuss the various configurations of the ES technique used for the manufacturing of polymeric MPs.

### 2.1. Types of Configuration

**Figure 2:**
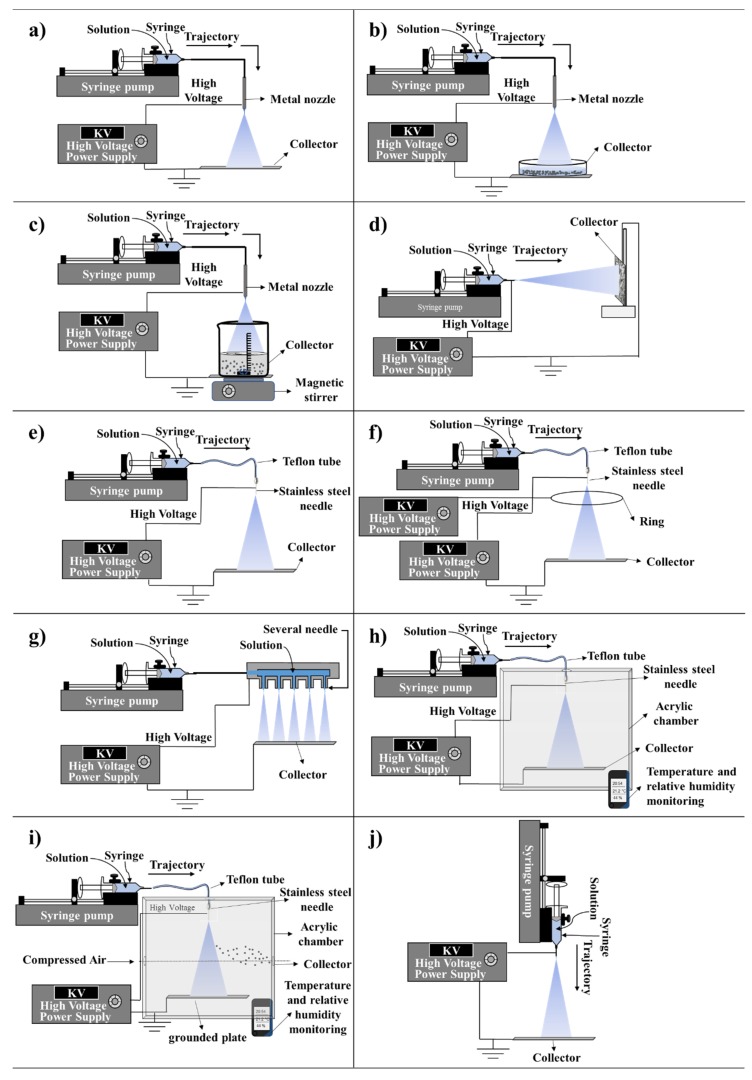
The various configurations applied for the ES technique for the production of MPs. (**a**) The simplest ES setup consisting of a syringe pump connected to a capillary connected to a high voltage source and a collector to collect the MPs; (**b**) similar setup as (a) but with a different collector; (**c**) similar setup as (a) but with a different collector and a magnetic stirrer added; (**d**) similar setup as (a) but the syringe pump and collector are positioned horizontally; (**e**) similar setup as (a) but with an additional silicone tube transporter from the syringe to the needle; (**f**) similar setup as (e) but with an extra power supply and a ring connected to the high voltage; (**g**) a multiplexed ES (MultES) setting containing several nozzles; (**h**) an ES setting performed in a chamber providing control over the temperature and humidity; (**i**) ES setting similar to (h) but with additional horizontal airflow to promote the evaporation of an organic solvent; and (**j**) demonstrates an ES setting of vertical orientation in the absence of any tube transporter.

In this section, the different types of ES configurations, i.e., equipment and objects (e.g., infusion pump, needles, and syringes etc.), employed will be covered. The different types of configurations and setups for the ES technology are summarized in Figure 2. The simplest setup is depicted in Figure 2a, which consists of a syringe pump connected to a capillary further connected to a high voltage source and a manifold to collect the MPs. This simple setting requires only a few pieces of equipment and apparatuses and, therefore, is an attractive and cheap option.

For all the setups in Figure 2a,d,e, the tip of the syringe is directly coupled to a needle or Teflon tube which in turn is coupled to a stainless steel needle and capillary of different thicknesses [27,28,29]. The difference of the settings demonstrated in Figure 2e is the additional silicone tube from the syringe to the needle that functions as a transporter for the polymer solution. This was employed by Nath et al. for the generation of solid and low-molecular poly (D, L-lactic-co-glycolide) (PLGA) MPs using the ES method for bone regeneration [30]. The authors also performed in vitro tests demonstrating a 90% encapsulation efficiency of simvastatin (a drug enhancing bone regeneration) over three weeks [30]. Moreover, Sander et al. investigated the influence of doping with NaCl in the manufacturing of polyvinylpyrrolidone (PVP) MPs by ES and concluded that this doping does not improve atomization in terms of particle size. Moreover, no effect on the particle size distribution was observed [20]. Furthermore, through the connection of a teflon capillary, the location of the syringe pump in relationship to the collector could be facilitated (Figure 2e,f,h,i) [31]. 

To date, there are a number of object types that have been employed and presented in the literature to serve as contacts between the liquid that is expelled from the syringe with the applied load from the voltage source. Some reports present a variety of denominations for the metal tip which is attached at the source, including a needle [12,13], nozzle [29,30], and capillary [31]. For a better understanding of these materials a schematic representative is depicted in Figure 2 according to some reports optimizing the ES settings. Throughout this review, we will come across these various denominations per different literature reports. However, to be consistent, the needle is selected to represent the metal tip which is used to transfer the electrical charge from a high voltage source and polymer solution. More details will be discussed in Section 2.2 Types of needles.

Moreover, ES settings can be varied by changing the collector. There are several types of collectors used to obtain particles, such as aluminum plates [31,32,33], stainless steel plates [34,35], steel plates covered with foil [15,32] and the receiver solution [28,36,37,38,39], which are all demonstrated in Figure 2a–d. Moreover, previous studies have demonstrated the use of alcohol solutions, buffers and surfactants as the MP receptor solution [28,36,37,38,39].

The jet formed during the ES process can be configured so that the jet can be expelled horizontally or vertically triggering the particles to flow and reach the collector in different orientations. As illustrated in Figure 2, some ES configurations are placed in the vertical orientation, which makes it easier for the collection of the MPs, compared to a horizontal jet which might limit the use of liquid collectors. Figure 2d depicts an ES technique in which the syringe pump and the collector are positioned horizontally. On the other hand, Figure 2j depicts an example of vertical orientation, representing a setting in which there is no tube transporting the polymer solution between the syringe and the needle. The system is made in a simple way where the infusion pump is horizontal or vertical of the collector [29,40,41].

The horizontal orientation was employed by Hao et al. who prepared porous MPs of PLGA dissolved in the solvent dichloromethane (DCM), loaded with the drug metronidazole (MTZ) through deposition of ES in a single step [40]. MTZ-loaded PLGA microparticles were characterized by Fourier Transform Infrared Spectroscopy (FTIR) and the authors observed that the characteristic bands of MTZ were present. However, no new bands were observed, therefore, the authors concluded that there was no polymer-drug interaction. Cell viability in the presence of the MPs was greater than 80%, which indicated that the cytotoxicity of the MTZ-loaded porous PLGA microparticles were non-cytotoxic against GES-1 cells for 12, 24, 36 and 48 h with concentrations from 500 to 800 μg/mL. The average size of the MPs obtained ranged from 1.29 ± 0.01 to 8.39 ± 0.10 dm and displayed a sustained release in simulated gastric fluid. Moreover, Bock et al. employed the horizontal setting for the encapsulation of vascular endothelial growth factor (VEGF) and bone morphogenetic protein 7 (BMP-7) in MPs from PLGA dissolved in chloroform and DCM, manufactured by ES, and further evaluated its activity for bone tissue engineering [41]. MPs made of PLGA and poly (ethylene glycol) (PEG) (PLGA:PEG) loaded with serum albumin at 1% by weight without the addition of trehalose in the initial solution were observed with an average size of 5.0 ± 1.3 μm and with the addition of 1% trehalose 5.7 ± 1.6 μm, respectively. This study demonstrated how MPs generated through ES ensured efficient delivery of fully active growth factors, relevant for the engineering of bone tissue.

In a report by Mouthuy et al., an exception to the ES setting in the horizontal or vertical arrangement of the needle was demonstrated. The authors aimed to manufacture a portable ESP and ES powered by a battery [28]. The equipment was processed in different directions due to the connection of a pen extension. Moreover, its versatility was demonstrated during the production of both filaments and MPs, and further showed the possibility of ESP directly on the skin. To date, there are no reports of comparative studies in the production of MPs with the ES system with a vertical or horizontal orientation. In this context, an advantage of the vertical device is the possibility of using collectors containing a solution. This can be very interesting to demonstrate experimentally, since the changes in the formation of the MPs manufactured in the vertical setting versus in the horizontal might differ significantly.

Moreover, Figure 2f demonstrates the ES technique in which a ring is connected to a high voltage or when a grounded source is employed. This setting is used to converge the droplets produced to the center of the spray for better particle collection, providing an advantage over the other ES configurations [42,43,44]. This setting was employed by Jafari-Nodoushan et al. who investigated the effects of FR, collection distance, molecular weight of the polymer, and needle diameter in the ES technique using PLGA with 52 variations of conditions involving solvent, FR, collector distance and needle gauge [44]. It has been shown that the morphology of the MPs can be modulated and their mean diameters can be varied from 10 μm to 43 μm by varying these parameters. There are not many studies using this ring configuration, therefore, it would be highly valuable for further investigations of this technology. For instance, some suggestions on further studies that might be of interest include the investigation on the variation of the distance between the ring and the tip of the needle and the variation of the ring radius and the thickness of the wire that forms the ring that is commonly made of copper.

The multiplexed ES (MultES) setting is illustrated in Figure 2g. Here, Almería et al. developed a MultES method containing several nozzles in which the solution is electro-vaporized, providing good control to generate PLGA based MPs with different morphologies by ES drying [45]. The authors obtained greater yield through the ES technique compared to the conventional ES [27]. The typical yield in the production of the PLGA MPs is around 0.01 g/h. Almería et al. overcame this limitation by operating multiple power sources in parallel using a microfabricated MultES device that resulted in particle production rates exceeding 10 g/h [27]. A further interesting study would be the merging of the MultES technique with the ring technique in Figure 2f, since there exists no such investigations to date. Therefore, it can be observed that the amount of MPs manufactured by ES is still a limitation compared to other technologies. Here, Almería et al. overcame this limitation by using the MultES methodology [27]. It could also be observed that the presented reports do not address mass quantities or the volumes of MPs obtained at the end of the process, which would be of great interest for the scientific community, which we hope will be addressed in the future.

Figure 2h demonstrates an ES setting performed in a chamber, which was employed by Bussano et al. [31]. The objective of their study was to evaluate the potential of the ES for the encapsulation of insulin in lipid particles using ES in a transparent closed box, which was used to control the temperature of 20 or 40 °C in which the MPs were designed [31]. Moreover, Almería and Gomez settled the ES source inside a camera [45]. The authors developed a methodology to control the size of the PLGA polymer particles through ES for biomedical applications. Furthermore, they could produce biodegradable PLGA micro or nanoparticles of uniform size using the drying route, i.e., employing vertical air co-flow. The neutralized droplets were carried by gentle air flow towards the collector since they would not anymore respond to the electric field. The average particle size obtained had an extremely wide range from 60 to 2000 nm.

Moreover, Yao et al. described ES produced in a chamber with horizontal airflow combined with a ring located near the needle (Figure 2i) [16]. A cross-flow of inert gas was used inside the chamber to aid in the evaporation of the organic solvent from the droplets and to collect the particles, a method known as pneumatic conveying [16]. A device was connected to control the temperature and humidity. The transport chamber also aimed to provide an isolated environment, avoiding undesirable external contamination [16].

To conclude, the setting of the ES apparatus can be constructed in several ways, showing that in theory, the researcher’s creativity and knowledge in the area of interest have a great impact when it comes to developing suitable materials. Here, it was observed that some studies were concerned about the interference from the external environment in the manufacturing of MPs when employing ES technology inside a camera [16,31,45], compared to recent studies, which have opted not to use the camera [20,39]. From this comparison, one might conclude that there is no necessity for using a camera for controlling the temperature and humidity of the medium in order to obtain MPs. Furthermore, the ES technique can be classified, based on the type of needle used in the process, such as, monoaxial, coaxial and tri-axial which will be discussed in the next section.

### 2.2. Type of Needle

#### 2.2.1. Mono-Coaxial ES (MES)

**Figure 3:**
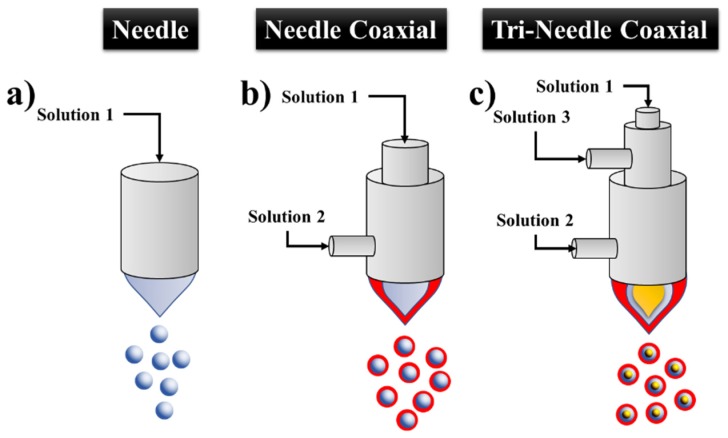
Representative figures of the various needles in ES technology: (**a**) simple needle, (**b**) a coaxial needle, and (**c**) a triaxial needle.

In this section, as well as the following two, we will explain what the settings MES, Coaxial ES (CES) and Tri-axial ES (TES) mean. Figure 3 depicts the various settings and demonstrates what a needle tip, nozzle, or capillary look like. However, for simplicity, we will use the term needle for these metal tips following the report from Steipel et al. in which they report a representative figure of these metal spikes similar to that suggested in Figure 3 [46].

In the monoaxial ES there are three types of needles used to design MPs. Figure 3a shows a simple needle while Figure 3b illustrates a coaxial needle which has another needle on it. Moreover, Figure 3c shows a tri-coaxial needle, which has three needles with one on the outer part, one needle in the middle part, and another needle in the inner part.

The technique that uses a single-hole needle is called MES or conventional ES, which consists of a single needle (Figure 3a) [30,31,37]. Moreover, Suksamran et al. designed MPs by using MES in which the orientation of the jet was vertically oriented (Figure 2j) [36]. The syringe pump was placed above the collector, a solution of calcium chloride (CaCl_2_) was used as the collector, and the setting employed was a combination of the setup presented in Figure 2b,j. An important detail in this study is that no device was connected to control the temperature and humidity, which could be a problem with this type of configuration. Additionally, Nath et al. designed MPs by MES using PLGA at different concentrations and the solvent DCM, which was further employed for the encapsulation of simvastatin [30]. The FR maintained was 0.50 mL/h, the inner diameter of the needle was 0.55 mm, the external diameter was 0.80 mm, the tension at 15 kV, the collector was stainless steel metal, and the average MP diameter obtained ranged from 1.6 to 3.963 μm. The MES technique must be carried out very carefully since designing polymeric MPs to encapsulate bioactive agents can cause damage to the agents when the polymer solution is added. For instance, using solvents to dissolve the polymers may cause degradation of the bioactive agent used. The initial in vitro studies (such as cell viability assay using osteoblast cells) demonstrated good biocompatibility and the gene expression analysis showed that the microspheres upregulated the gene expression for extracellular matrix proteins promoting bone formation [30].

#### 2.2.2. Coaxial ES (CES)

The coaxial needle has an inner and an outer needle, as demonstrated in Figure 3b, and the technology is called CES [47,48]. This approach is commonly used to design polymeric MPs which can encapsulate a filler, for example, proteins [42,49], living yeast cells [50], and drugs [12,13,29,40]. In CES, two immiscible liquids or a liquid and a gas are injected into the outer and inner needles, with FRs controlled by two syringe pumps, respectively. By applying a high voltage, varying in tension for electric field formation and employing an appropriate FR, a Taylor cone is formed and later the internal and external liquid is eventually broken into multilayer droplets. Finally, the droplets are collected by a grounded electrode or a container underneath the electrode [51].

Funasaki et al. used the CES setting to design hollow giant lipid vesicles by a single step coaxial electro-emulsification method, having separate solutions of a phospholipid and a degradable polyelectrolyte [35]. The CES has an internal needle with internal and external diameters of 330 and 630 μm, respectively, and an external needle with diameters of 1.0 and 2.5 μm, respectively. The internal solution flow was fixed at 1.0 mL/h and the external flow was set at 0.5 mL/h. A poly (β-amino esters) (PBAE) solution was placed in the inner part and the PEG solution was placed on the outside of the coaxial system. The same year, Liu et al. developed solid dispersions (SDs) in the form of core-shell MPs with mean diameters of 1.36 ± 0.67 and 1.74 ± 0.58 μm [12]. The authors employed the CES technique and used PVP as the hydrophilic polymer matrix and acyclovir (ACY) (an antiviral agent) as a poorly water-soluble model drug. Perceptibly, CES has certain advantages over MES, where one of the advantages is the production of MPs with polymeric coatings [35]. In this context, Funasaki et al. employed this strategy, where the poly(β-amino esters) (PBAE) was employed as the core, and PEG as the shell, which would not be possible by the use of the MES approach [35]. A further advantage was demonstrated by Liu et al., where acyclovir (ACY) was encapsulated into PVP ensuring that in the core, the drug was protected by a polymeric shell [12]. This would not be possible by MES, since it lacks the core-shell encapsulation capacity, which results in the dispersion of the drug between the polymeric structure [12]. A further advantage of CES in comparison to MES is that it allows for the preparation of the solutions separately. In this context, it is important considering that the solvent used to dissolve the polymer might also result in degrading the drug or bioactive agent. Therefore, CES methodology allows for the preparation of the drug solution separately by using the appropriate solvent, for example water, and in parallel, the polymer solution with a suitable solvent, such as chloroform. In this way, the drug can be preserved since there is no need to place the drug in the same polymer solution as in the case for the MES approach. Nevertheless, there are some challenges encountered in forming consistent two-tier constraints. One of the challenges is the regulation of the usage parameters, because in the case of CES, the process proceeds by using two FRs, one for an internal solution and the other one for an external solution that must be adjusted to form a stable Taylor cone. An additional challenge is related to encapsulation damage such as electrochemical damage, drop charges, drying of the droplet, the landing impact of the charged molecules on the grounded collector, and the ambient and environmental conditions [52].

#### 2.2.3. Tri-Axial ES

The tri-axial ES technique (TES) as demonstrated in Figure 3c uses three needles: an external, central and internal needle. This technique has also recently been employed for the design of multilayer particles for the fabrication of magnetic polymer yolk-shell particles (YSPs) using a single-step coaxial three-needle TES technique [53]. The YSPs allowed for the hosting of multiple probes (as chemical modeling agents) for dual-mode imaging (magnetic and ultrasonic resonance imaging) with specific multi-drug compartments through an advanced single stage encapsulation process. Further biological investigation of the YPS demonstrated a low cytotoxicity (5 mg/mL) and with good biocompatibility found for murine L929 cells. The tri-axial needle setting (Figure 3c) enables the formation of 3-layer particles, which is different compared to the CES and MES approaches. Moreover, TES allows for the creation of more needle configurations with higher numbers of capillaries, which is a needle with four coaxial needles on it.

### 2.3. Multi-Capillary ES (MCES)

The multi-capillary ES (MCTES) section was selected to be separate from the type of needle section in order to avoid any confusion in understanding the concepts, as the TES setting consists of a side-by-side multi-capillary structure. A ES system comprised of a combination of several capillaries arranged in parallel is called multiplexed ES or MultES and has the potential to increase the yield of MPs manufacturing under large scale production (Figure 2g) [27]. Almería et al. employed a microfabricated MultES device consisting of a silicon spike chip and an extractor, in which the chip was several ES nozzles arranged in a hexagonal pattern [27]. Moreover, they used two types of chips, one with 7 sources and another with 19 sources with internal and external diameters of 210 μm and 60 μm, respectively. The MultES method allowed for the fabrication of PLGA MPs with different morphologies. The final morphology was based on the sequence of Coulomb fission and the entanglement of polymers, which can be controlled by selecting the proper molecular weight of the polymer, the concentration, and the FR of the solution [27]. 

### 2.4. Experimental Parameters that Influence ES During the Design of Microparticles

In the previous sections, pertinent fundamental concepts were highlighted for the ES setup, but here, several parameters that influence ES to obtain desirable final MPs will be discussed.

The basic principles of the ES technique are similar to those from ESP to design the filament. Depending on the concentration of the polymer used during the ES process, particles or fibers can be obtained [54]. ES is useful to design nano or MPs. This size variation can be obtained by controlling experimental parameters, such as FR, needle diameter and applied voltage, as well as chemical composition and the concentration of the sprayed solution [12]. Other crucial parameters influencing ES technology are: polymer concentration, solvent employed, applied voltage between needle and collector, distance between nozzle and collection plate, type of manifold, FR, type of syringe, ES chamber, temperature, relative humidity (RH), and caliber of the needle (Table 2). Depending on the application of the produced particles, these parameters can be adjusted conveniently allowing for the fabrication of particles with different shapes and sizes, suitable for a wide range of applications.

#### 2.4.1. Polymer Concentration

In this section, we will discuss the influence of the polymer concentration on the outcome of the MPs and the ES process. As highlighted, the polymer concentration is an important parameter and, in this context, the configuring of the ES technique is practically identical to the ESP technique. The basic difference between these techniques is the molar mass and the polymer concentration being employed. High concentrations of the polymer lead to a trend towards yarn formation, as demonstrated by Wang et al. in which high polymer concentrations of poly(methyl methacrylate) (PMMA) dissolved in DMF were tested [15]. This resulted in the formation of fibers rather than particles, as would be expected from the ESP technology. However, as the PMMA concentration decreased, the authors noticed a change in the morphology of the material to a spherical shape. Thus, the polymer concentration plays an essential role in directing the technique to either ES or ESP [15].

Moreover, Correia et al. designed poly(vinylidene fluoride) (PVDF) based MPs by varying the concentration of the polymer solution from 2% to 10% (*w*/*v*) [10]. They observed the formation of fibers as the concentration increased. In addition, interestingly, the shape of the MPs was spherical at a 2% concentration and at a 7% concentration it was possible to detect the formation of fibers. From the 10% polymer solution, despite the presence of MPs obtained from the technique, there was a substantial increase in the amount of fibers obtained. Several groups have demonstrated the tuning of the polymer concentration for the generation of MPs, for instance Faramarzi et al. employed PLGA at a polymer and solvent ratio of 2% (*w*/*v*) and Sander et al. used PVP at a ratio of 5% and 10% (*w*/*v*) for the generation of MPs, respectively [20,39]. 

The influence from the polymer concentration on the obtained morphology is related to the viscosity of the polymer solution, where a higher concentration leads to an increase in viscosity. Since electric fields are applied in the ES systems, when it exceeds a critical voltage, the electrostatic forces overcome the surface tension and force the ejection of a liquid jet. In contrast, in low viscosity solutions, the jet is divided into droplets, which is the basic principle of ES. For high viscosity solutions, the jet does not break, but rather transports as a jet to the grounded target (ESP), which is used to make polymeric microfibers or nanofibers. Therefore, relatively dilute solutions are necessary in order to avoid fiber formation [15,31,45].

**Figure 4:**
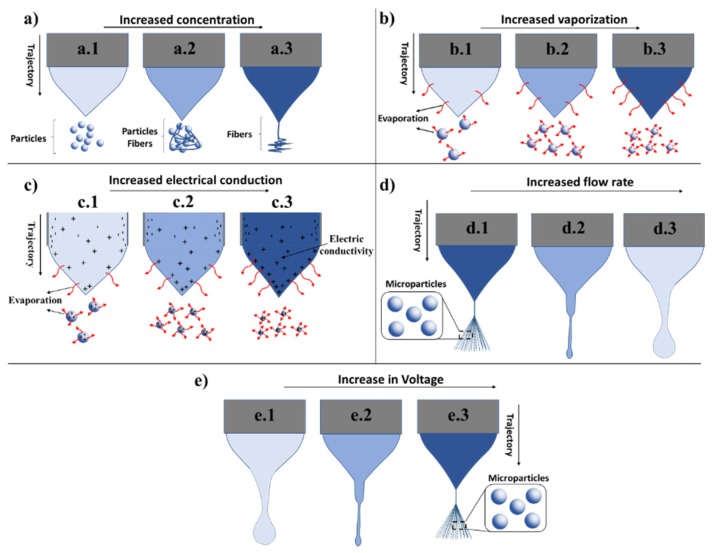
The impact on ES performance and MP size from various parameters. (**a**) The influence from changing polymer concentrations: (**a.1**) low concentration, (**a.2**) medium concentration and (**a.3**) high concentration. From left to right the concentration of the polymer increases. (**b**) The effect of solvent volatility: (**b.1**) low volatility, (**b.2**) medium volatility, and (**b.3**) high volatility. Increased volatility from left to right. (**c**) The influence of the electrical conductivity of the solvent: (**c.1**) low conductivity, (**c.2**) medium conductivity, and (**c.3**) high conductivity. Increase in electrical conductivity from left to right. (**d**) The impact of changing flow rate (FR): (**d.1**) low FR, (**d.2**) medium FR, and (**d.3**) high FR. The FR increases from left to right. (**e**) The influence from tension. (**e.1**) low voltage, (**e.2**) medium voltage, and (**e.3**) high voltage. The applied voltage increases from left to right.

According to previous reports, the importance of the parameters for mass and volume during the preparation of the polymer solution for the ES are eminent for the final products, and in general, a ratio between 2% and 10% (*w*/*v*) are recommended for obtaining MPs [10,12,20,39,40,55]. Within this framework, Figure 4a illustrates how the concentration of the polymer would impact the outcome of the ES process and generate products, where low polymer concentrations (well-diluted solutions) would provide MPs (Figure 4(a.1)). In the case of higher polymer concentrations providing higher viscosity (solution concentrations at 10% (*w*/*v*)), sufficient surface tension favoring the entanglement of the polymer chain and favoring the formation of fibers between the MPs would result (Figure 4(a.2)) [10]. In the case of higher concentrations (higher than 10% (*w*/*v*)), the formation of fibers is favored as illustrated in Figure 4(a.3). In all the scenarios presented, the solutions are composed of only the polymer and the solvent and, therefore, in the absence of any dopants or charge to impact the fabrication of the particles, these will be discussed in the following sections.

#### 2.4.2. Type of Solvent

The choice of the right solvent is crucial, both for the fabrication of the MPs but also for their applications in various biomedical challenges [39]. Parameters and physical properties of the solvent that influence the ES processes are electrical conductivity, surface tension, viscosity, dielectric constant, and evaporation rate [30,39,49].

The choice of the solvent is based on their volatility properties. Generally, the ideal preferable choice for ES particle design are solutions of low molecular weight polymers dissolved in low volatility solvents [45]. Moreover, the evaporation of the solvent is another important parameter that must be taken into account since it has a high influence on both size and morphology of the generated particles [30,39,44]. One of the effects of choosing a solvent with a low evaporation rate is the bonding and agglutination of the collected particles [16]. Solutions prepared containing other components than the polymer, such as the drug, require attention since the solvent employed to dissolve the polymer might lead to damage to the drug or the encapsulation of the drug [56].

In the ES technique, the evaporation of the solvent is induced by the tension applied at the tip of the needle and, depending on the volatility, the solvent can regulate the size and morphologies of the produced particles [39,44,45]. If the solvent employed does not evaporate completely before the collector, the particles may become wet and subsequently infer on the biomedical applications, since the solvents used can cause cellular damage [16].

In this context, Duong et al. investigated the production of MPs made of dextran acetal polymer (Ac-DEX) in six different solvents: trifluoroethanol, methanol (MeOH), ethanol (EtOH), 1-propanol, 1-butanol and 1-pentanol [34]. The choice of these solvents was based on the solubilization capability of Ac-DEX and their volatility changed with chain length and halogenation. The trifluoroethanol, MeOH, EtOH, 1-propanol, 1-butanol, and 1-pentanol had a mean particle diameter of 1.3 ± 0.2, 1.0 ± 0.3, 2.5 ± 0.5, 2.3 ± 1.2, 2.6 ± 1.5, and 2.2 ± 1.4 μm, respectively. These results confirmed that the solvent influences the size of the particles. The authors selected EtOH as the optimal solvent for further studies due to its less toxic nature compared to other solvents commonly used, such as DCM and chloroform. In addition, the residual elimination of such solvents after the MPs are fabricated, is favorable and there is a greater interaction of solubility between EtOH and the polymer acetalated dextran (Ac-DEX) compared to PLGA and other polymers of polyester origin [34].

For the CES process, Duong et al. used an aqueous phase in the outer needle in order to avoid the evaporation of the chloroform; it was placed in the internal phase of the droplet [38]. The role of the external aqueous phase of the droplet was to avoid significant evaporation of the organic phase at a transit time from 30 to 50 ms, which corresponded to the exit of the jet to the collector plate. Thus, extensive knowledge of solvent and non-solvent properties in the ES system is important [38].

In Figure 4b, the importance of solvent volatility is demonstrated where an increase in volatility resulted in an increased rate of evaporation and, therefore, provided particles with smaller sizes. Figure 4(b.1) depicts MPs obtained from solvents with a low evaporation rate, Figure 4(b.2) with an average evaporation rate and Figure 4(b.3) with a high evaporation rate, respectively. 

#### 2.4.3. Solution Conductivity

Solvent conductivity is another important parameter for the ES system and will be highlighted next. The electrical conductivity of the solution can be used to control particle size. This parameter can be increased through the addition of a small amount of dopants into the solvent. To solve this, Almería and Gomez employed triethylammonium formate (TEAF) (at concentrations ranging between 25 μL/mL to 100 μL/mL) as a dopant to increase the electrical conductivity of the solvent solution (for 25 μL/mL, the conductivity was 1202.64 μS/cm and for 100 μL/mL, 4871.45 μS/cm, respectively, and for dimethylsulfoxid (DMSO) and NaCl (2.74 mg/mL)) [45]. 

Moreover, Duong et al. observed that the addition of the Tween 80 surfactant to the formulation changed particle morphology. The authors concluded that this surfactant and the other used (Tween 20) increased the conductivity of the solution to the same order of magnitude, which contributed to changes in morphology of the particles with a reduction in size of the primary drops. However, the authors failed to explain the observed discrepancy and instead affirmed that new studies which included better characterization of the physical properties of the particles would be necessary to explain the differences observed [34].

Gallovic et al. employed CES and observed some difficulties in keeping a stable Taylor cone. This challenge was solved by employing a buffer as the internal solution used to prepare a recombinant protective antigen (rPA) solution to reduce the conductivity of the solution [56]. Sander et al. carried out ES to generate PVP MPs from polymer solutions and investigated the influence of adding an electrically conductive dopant [20]. Experiments were conducted to determine the effect of Na^+^ and Cl^−^ ions at concentrations ranging from 0.006 to 0.048 wt %. The authors observed that adding these elements allowed one to increase the FR and polymer concentration to improve particle yield. In contrast to theoretical scale laws, a four times increase in conductivity (from ~50 to ~200 μS/cm) through the addition of ions did not cause significant effects on particle size. The authors concluded that doping with Na^+^ and Cl^−^ ions cannot be used as a method to increase the production of PVP particles by ES [20]. Figure 4c demonstrates the effect of increased electrical conductivity of the solvent on ES technology and particle morphology. With increased electrical conductivity, the rate of the evaporation increased, which in turn led to the generation of particles with smaller size. As noted, there are controversies in increasing the solution conductivity, nevertheless we should be aware of such properties in each case. As previously discussed, Sander et al. showed that quadrupling conductivity did not improve atomization in terms of particle size [20]. Previous studies have already confirmed through observations that the addition of a dopant interferes in the manufacturing process of MPs [20,34].

Based on all of the above observations, it can be concluded that MPs can be obtained by tuning experimental parameters that control organic solvent evaporation rate. For instance, Xie et al. investigated and controlled the variations of MP morphology by varying the polymer concentration (PLGA). The authors observed that as the PLGA concentration increased, the MPs formed smaller agglomerates with a smoother surface [18]. Here, PLGA concentrations below 4% (*w*/*v*) provided MPs with morphologies ranging from small fragments to spheres with high porosity. These particles had a very large surface/volume ratio, with their degradation occurring at a relatively rapid rate. Conversely, at concentrations higher than 4% (*w*/*v*), the formation of more spherical particles with lower porosity was observed. These smooth surface MPs had a lower surface/volume ratio and therefore a slower degradation rate compared to the aforementioned MPs. In this context, porosity control is important since this can generate MPs for medical applications. For example, particles with higher surface porosity may be used in drug delivery due to their large surface area and low density. Emphasizing that pore formation can be controlled by changing the organic solvent evaporation rate using different experimental configurations or air FRs is another important finding. For example, in this study, the authors employed three different types of settings (setting 1: EHDA without ring; setting 2: with a ring and setting 3: with a ring and a chamber). Uniform MPs were produced when using setup 2 compared to setup 1. Moreover, particles of medium size (255 nm) were obtained by setup 3 and these were slightly smaller than those produced by setup 2 (355 nm). In addition, the authors observed that particles with a more homogeneous morphology were obtained using setup 3. The authors concluded that differences in solvent evaporation rate may have contributed to morphological differences achieved as the solvent evaporated faster using setup 2. The concentration of organic solvent in the vicinity of the spray cone of setup 2 was very low as it sprayed directly into the ambient air, which acts as a sink for the evaporated organic solvent. In other words, high solvent concentrations would most likely result in a slower evaporation rate. Therefore, more studies need to be performed concerning the influence of the dopant on the production of MPs.

#### 2.4.4. Flow Rate (FR)

Here, we will discuss one more parameter that effects the manufacturing process of MPs, which is the FR. The device used to control the FR of the solutions located at the tip of the needle is called a syringe or infusing pump. The FR is a parameter that can influence the morphology of the particles providing porous or smooth beads, and further impacts the size distribution of the MPs [16,55]. A stable Taylor cone jet shape can be obtained through appropriately adjusting the FR (Figure 1) [38,49].

Prominently, the volatility of the solvent has a direct impact on the FR, since at the stage when the solvent leaves the needle tip, there is a process of evaporation induced by the applied tension. This process influences the size of the jet leaving the needle; therefore, it is important to have the right balance between the volatility and FR. As previously mentioned, the concentration of the polymer is also an important parameter, which favors the formation of MPs instead of a jet as in the ESP [45]. Here, the FR will determine the amount of polymer solution available for ES, where high FR can result in impaired total evaporation of the solvent during the transportation of the particles from the tip of the needle to the collector. These can further lead to partially solvated MPs, and therefore, with deformed and non-consistent morphology [10]. Figure 4d demonstrates the behavior of a polymer solution outlet at the tip of the needle when the FR is increased. Within this topic, Yao et al. demonstrated that a low FR led to the formation of dry particles and that a high FR provided moist particles [16]. This is explained by the fact that the droplets are larger, which increases the evaporation time required, which in turn could be affected by the distance between the needle tip and the collection plate. However, if the distance between the nozzle and the grounding plate increases, the electric field decreases causing the droplet size to increase. Moreover, the increase of the applied voltage leads to a decrease in the average radius of the droplet size, therefore depending on the distance, the voltage will have to be adjusted so that the electric field is at an ideal level [16]. Within this topic, Bock and coworkers observed a decrease in particle size due to the addition of PEG to a solution of polycaprolactone (PCL) (10% *w*/*v*); these lead to problems with the encapsulation of large molecules [42]. This limitation was surmounted by increasing the FR 6 times, providing larger MPs with a reproducible process. For example, in this study, several proteins were successfully micronized as spherical MPs with diameters of less than 5 μm. The addition of PEG in different proportions (PEG:protein) showed great efficiency in controlling the size of protein aggregates. These protein aggregates decreased in size with increasing PEG concentration in the proportion previously mentioned, until a critical point was observed. This made it possible to decrease the size of the protein spheres more slowly and in a controlled manner. In other words, the addition of PEG to a PCL polymer solution led to instabilities in the ES, which in turn generated non-uniform ES particles to a larger extent. Moreover, Correia et al. investigated the influence of the FR on the size of MPs of PVDF [10]. When a FR of 0.2 mL/h was employed, MPs with smaller mean diameters were obtained compared to when higher FR where used, providing particles with larger average diameters. The increase in diameters is a result of a competitive mechanism of the Coulomb fission, where the drop of charge emits small highly charged vaporized droplets as a result of saturated charges within the droplet (the Rayleigh limit) [10]. Moreover, higher FR (>4 mL/h) resulted in wet deposited MPs, due to the lack of sufficient time to evaporate during the process since greater amounts of the solution were ejected. These findings also gave rise to significant particle aggregation when reaching the metallic ground collector.

Xie et al. presented several solutions to overcome the problems associated with obtaining a stable CES. In their aim, to obtain a stable CES, they employed a lower FR for the inner protein solution compared to the external solution [49]. They also highlighted that in order to obtain MPs with greater amounts of drugs loaded, it is enough to increase the FR or the concentration of the drug in the internal solution [49]. In fact, the Coulomb fission and polymer entanglements can be controlled through several strategies, such as tuning the FR, the molecular weight of the polymer, and the concentration and conductivity of the solvent [39]. Here, Faramarzi et al. observed that the morphology of the MPs could be changed from a cup to spherical shape or to a concave morphology by altering the FR from 0.2 and 0.3 to 0.5 to 1.0 mL/h [39]. Further, increasing the FR (2.0 mL/h) resulted in deformation and an inconsistent morphology. Thus, they demonstrated that at high FRs, the solvents do not have sufficient time to be removed from the droplet causing partial evaporation of the solvent [39]. Figure 4d illustrates the impact of gradually increasing the FR in the ES technology, where it can be clearly observed that an increased FR leads to accumulation of the polymer solution, which impairs the evaporation essential for the formation of MPs.

#### 2.4.5. Electric Field

This section will cover the electric field parameter and its impact on ES technology and on the generated MPs. The ES process only starts when a voltage is applied [10], depending on the formulations, the voltage can vary from 2.5 to 30 kV [15,31,41,57,58]. Correia et al. investigated the effect of the electric field on designing PVDF MPs. The authors used electric hoods ranging from 0.75 to 1.25 kV/cm. They observed that when an electric field larger than the chosen one was applied, there was a tendency of forming MPs or elongated fibers. In this case, they concluded that lower electric fields, between 0.75 to 1.25 kV/cm, stabilize the main drop and the ejection of smaller jets which led to PVDF MPs with a larger size distribution [10]. Moreover, Xie et al. studied dripping with or without an electric field [49]. They found that when the solution left without an electric field, the drip was driven only by gravity and as the electric field was applied, the drip was influenced by the field and the droplets became smaller [49]. Therefore, the electric field allowed for the formation of a stable Taylor cone jet which divided charged vaporized droplets and formed MPs after the evaporation of the solvent from the fine droplets [49]. An illustration of a stable Taylor cone jet in MP design can be seen in Figure 4e in which the applied electric field increases from right to left.

Faramarzi et al. observed that the size of the MPs decreases with increasing voltage [39]. High or low voltage led to jet instability and the formation of irregular particles with a large size distribution. Therefore, it is of the utmost importance to find out at what voltage the most stable jet is produced. Experiments carried out with 2% (*w*/*v*) PLGA solution, a FR of 0.5 mL/h and applied voltages of 11 and 14 kV led to more spherical particles compared to the ones obtained when applying higher voltages which led to heterogeneous morphologies. In order to obtain more uniform particles, the authors selected a voltage of 11 kV, since they observed that when the voltage increased, the shape uniformity diminished [39]. Therefore, the applied voltage has a significant influence on the morphology, the mean diameter, and the uniformity of the MPs. An increase of voltage also led to an increase in solvent evaporation (Figure 4e).

#### 2.4.6. Temperature and Humidity

The impact from temperature and humidity factors on ES and the generated MPs will be discussed in this section. Bock et al. employed the ES technique to design MPs with dry encapsulated proteins using PEG (as the micronizing and solubilizing agent) merged with polycaprolactone (PCL) and PLGA [42]. The temperature and RH ranged from 23 to 24 °C, and 34% to 49%, respectively. In their studies, the influence of temperature and humidity were not addressed and in fact other authors do not either discuss the importance of temperature and RH in the ES process [42]. Moreover, Wang et al. developed PMMA MPs dissolved in DMF and ES at 22 °C with 32% RH [15].

Further, Liu et al. carried out the ES processes under ambient conditions (22 ± 3 °C, RH of 52 ± 6%) for the generation of core-shell solid dispersions of acyclovir (ACY) [12], and correspondingly Hao et al. employed similar conditions for the development of Eudragit® RS (ERS) porous MPs loaded with MTZ [55]. Porous PLGA based MPs have also been developed under ambient conditions [40]. Interestingly, Jafari-Nodoushan et al. investigated 52 different conditions for the production of MPs by varying polymer concentrations, polymer combinations, solvent, FR, collecting distance, and needle gauges (G), and the temperature employed was 25 °C and RH of 40% [44]. However, the authors did not provide any detailed information on the impact from the temperature and humidity, which would be of significant importance since these parameters are essential for the reproducibility of the procedure. Nevertheless, Correia et al. stated that the moisture present in the atmosphere contributed to the high surface roughness of their MPs obtained from PVDF in a DMF: tetrahydrofuran (THF) (DMF:THF) solvent mixture [10]. In fact, it is recommended that the particles should be kept away from sources of moisture, such as respiration, as they are easily damaged [20]. Here, Dai et al. designed particles by CES, in order to effectively control the stability of the system and eliminate impacts from the external environment, the conditions employed included room temperature (25 ± 5) °C and RH (30 ± 5%), and without having rapid air flow [48]. Correspondingly, MPs made of PLGA and by the employment of acetonitrile (ACN) and DCM as the solvents under similar conditions have been disclosed [16]. It was noticed that most of the PLGA/DCM particles were obtained with a spherical morphology and smooth surface, whilst the PLGA/ACN particles displayed a less spherical morphology. Fascinatingly, this phenomenon could be explained due to the different cooling effect on the solvent impacting the evaporation where a strong cooling effect resulted in lower evaporation rates and DCM displayed a stronger cooling effect compared to ACN. Therefore, the temperature at which the experiment is conducted should be considered as an important parameter since depending on the solvent used, it can lead to a low rate of evaporation and cause the collected particles to bind, unite, and agglomerate [16].

Furthermore, thermosensitive MPs composed of poly(N-isopropylacrylamide) (PNIPAAm)/phosphatidylcholine (PC) have been produced by ES at 25 ± 2 °C with 50 ± 5% RH [32]. According to the authors, the selected operating temperature was due to the fact that the MPs were thermosensitive. In their experiments, they analyzed the amount of drug release both at 25 °C and at 37 °C. Therefore, if the material responds to elevated temperatures, the ES system would necessarily have to be operated at lower temperatures [32].

Bussano et al. prepared a solution of 1-propanol containing palmitic acid (PA) or stearic acid (SA) at different concentrations [31]. The procedure was carried out in the presence of ethylcellulose or Pluronic F127 at two different temperature conditions (20 or 40 °C) in a closed transparent environment under magnetic stirring. Thus, by comparing the results obtained using the two temperatures, the authors chose PA due its higher solubility in propanol (about 12% at 20 °C and 25% at 40 °C) compared to SA (5% at 20 °C and 12% at 40 °C) [31]. From these presented reports, it can be argued that depending on the conditions in which the MPs are produced and which solvents are used, it is important to consider the two parameters: temperature and RH. Most ES methodologies operate at temperatures around 25 °C and at atmospheric pressure, which emphasizes that this technique is excellent for manufacturing nano or MPs under these conditions. Based on the reports to date, these parameters can be considered the least studied by researchers. Moreover, based on all of the above information, it can be noted that there are many parameters to consider which can be adjusted in the ES process. However, the most commonly tuned parameters when it comes to the manufacturing of the MPs are RF, the types of solvents and polymers employed. Hence, the temperature and humidity are limited only to stabilization prerequisites of the ES system, and in the small number of studies that even mention these parameters, they mainly solely state what the temperature and humidity values were without a further detailed investigation on the influence of these parameters on MP properties [44].

#### 2.4.7. Collector Distance

Additional parameters impacting the ES process and final MP properties include the distance between the collector and the needle tip. In ES, a high electrical potential difference at the kV scale is applied to the flowing liquid which is one of the parameters that can be easily adjusted. This is influenced by the distance between the tip of the needle and the collector, and this distance influences the properties of the MPs, such as size and evaporation rate of the solvent [30,59]. Here, the distance between the tip of the needle and the collector can be adjusted from 6 to 30 cm [28,29,31,36,57,60,61,62]. In this context, Faramarzi et al. observed that when designing PLGA MPs at distances of 4, 7, 13, 19, and 26 cm, as the distance increases to values greater than 13 cm, the size of the MPs decreased slightly to a diameter of 3.6 μm [39]. This was associated with the fact that longer distances would allow for more time for the solvent to evaporate. However, the authors observed that as the distance increased to 26 cm, the uniformity of the MPs decreased [39]. In contrast, PLGA based MPs also have been designed by varying the working distance to 3, 6, 9, 13, and 16 cm, setting the parameters of voltage (~10.5 kV), FR (2 mL/h) and needle size (21 G) [44]. In this report, it was detected that a distance of 3 cm did not provide spherical MPs, instead it formed aggregates due to the low evaporation of the solvent. At this distance, the time between the tip of the needle and the collector was not enough for the complete evaporation of the solvent. When the distance increased to 6 cm, they observed that the shape of the MPs was more spherical, but aggregated particles still formed. The authors further noticed that as the distance increased, the evaporation of the solvent was almost completed before the droplet reached the collection bath, which caused a difference in size and morphology of the particles. Thus, the flight time of the MPs increased by increasing the distance allowing for more time for the evaporation of the solvent which in turn decreased the size of the MPs [44]. Consequently, it is of the utmost importance to pay close attention to the distance between the collector and the needle tip.

#### 2.4.8. Types of Collectors

In this section we will discuss the various collector types and their influence on the ES manufacturing of MPs. In the ES process, there are several ways of collecting the MPs, such as on stainless steel plates [34], stainless steel plates coated with an aluminum foil [28], aluminum foils [40], an aluminum plate [63], beakers or Petri dishes containing different solvent such as MeOH or EtOH [39], Polysorbat 80 surfactant (Tween 80) [39], phosphate buffer or citrate buffer [35], solutions containing 2% (*w*/*v*) CaCl_2_, etc. [64]. In this context, Faramarzi et al. investigated the production of MPs of PLGA in different collection baths [39]. When the generated materials were collected in a solution containing a mixture of MeOH and EtOH, it was observed that the MPs had a concave shape, with elongated morphologies. As explained by the authors, EtOH and MeOH solubility parameters are close to each other, thus, providing particles with similar morphologies. It seems that the extension of time to complete the phase separation process affected the primary structure of the immersed particles [39]. When the particles were collected in a mixture of EtOH and saturated polyvinyl alcohol (PVA), a mixture of EtOH and Polysorbate 80 surfactant (1% *w*/*w*) and a mixture of EtOH and oleic acid, the particles had a spherical shape. Faramarzi et al. explained that the surfactant acted as a size and morphology stabilizer that surrounded the immersed particles and maintained the spherical shape of the sprayed droplets [39]. The type of ES methodology and the specification of the parameters used, such as FR, internal and external diameter of the needle, type of collector, working distance, applied voltage, temperature and RH, among others are listed in the supporting information (Supplementary Table S1). The distance between the collector and the tip of the needle, according to Supplementary Table S1, was between 5 cm as a minimum distance and 30 cm as the maximum distance. Another important fact that can be observed is that the applied voltage varied from 2.5 to 30.0 kV and the FR from 0.1 to 9 mL/h. The main solvents and polymers employed in the study are also presented as Supplementary Table S2. These polymers were PLGA, alginate, chitosan, PCL and PVA. Moreover, among the solvents presented in Supplementary Table S2, the most used are DCM, THF, DMF, water, chloroform, and EtOH.

### 2.5. Types of Microparticles Made by ES and Their Applications

**Figure 5:**
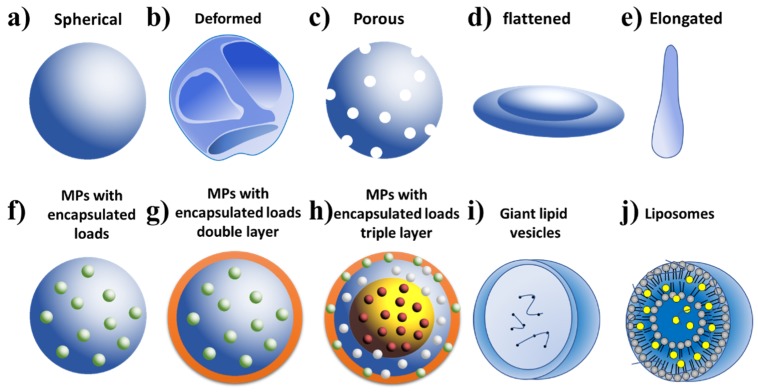
The different types of MPs generated from the ES technology: (**a**) spherical, (**b**) deformed, (**c**) porous, (**d**) flattened, (**e**) elongated, (**f**) MPs with encapsulated loads, (**g**) MPs with encapsulated loads with double layers, (**h**) MPs with encapsulated loads with triple layers, (**i**) giant lipid vesicles, and (**j**) liposomes.

In this section, various types of MPs that can be obtained as well as their medical applications will be discussed. Several forms of MPs can be obtained by the ES technique and some examples are presented in Figure 5. These could be spherical MPs [44], deformed/wrinkled [56], porous [30,39], elongated [16,29] and flattened shapes (morphology similar to red blood cells) [16]. Moreover, drug or biologically active agent encapsulated MPs can be obtained by using MES, CES, and TES technologies. The MES technique allows for the generation of MPs with only one layer. In the case charge is applied, the charge is distributed in the MP as demonstrated in Figure 5f [29,40]. When the CES method is used, depending on the settings, double layer MPs can be obtained with load located inside the MPs (Figure 5g) [12]. In addition, vesicles may also be produced (Figure 5i) [35]. The vesicles developed by Funasaki et al. through CES with internal needles displayed internal and external diameters of 330 and 630 μm and the external needle produced MPs with diameters of 1.0 and 2.5 μm, respectively [35]. 

Moreover, giant lipid vesicles can be generated by using a high voltage generator set at 23 kV coupled to the needle tip (cathode) and a collector with an oppositely charged stainless steel (anode) plate with a citrate buffer bath. The buffer solution was gently shaken with the aid of magnetic stirrer. The internal flow of the solution was fixed at 1.0 mL/h and the external flow was set at 0.5 mL/h. The poly(-β-amino esters) PBAE solution was placed in the inner part, and the PEG solution on the outside of the coaxial system [35]. In the study, four combinations of ES solutions and receptor solutions were examined. The vesicles were prepared with: (1) a 10% by weight PBAE/hydrochloric acid (HCl) solution (ES solution) and citrate buffer (receiving solution); (2) a 10% by weight PBAE/HCl (ES solution) and phosphate buffer (receptor solution); (3) a PBAE/acetate at 10% by weight (ES solution) and a citrate buffer (receptor solution); (4) a PBAE/acetate (ES solution) and a phosphate buffer (receptor solution). The ES of the PBAE/acetate solution in the phosphate buffer produced giant vesicles. On the other hand, the PBAE/HCl solution through ES technology failed to successfully generate any vesicles [35]. Phase contrast microscopy revealed the fruitful formation of a gel-like complex (PBAE/citrate ion). After 24 h, at room temperature, the gel-like complex disappeared almost completely, indicating that it degraded rapidly. These preliminary results suggested the potential of PBAE as a degradable model for preparing giant lipid vesicles by CES. 

Furthermore, liposomes can also be produced by ES (Figure 5j), which have been demonstrated by Jin et al. for use as a drug delivery system [32]. The liposomes were fabricated by dissolving different concentrations of PNIPAAm in PC, and in a chloroform/EtOH solvent mixture (1:1 *v*/*v*). Six different solutions (M_1_–M_6_) were prepared among which, 5 solutions contained a fixed PNIPAAm amount at 10% *w*/*v*, and the PC concentration ranged from 1 to 10% *w*/*v*. Moreover, the solution contained the drug Ketoprofen (KET) (a nonsteroidal anti-inflammatory drug (NSAID)) which was prepared at the ratio of PNIPAAm (10%)/PC (5%)/KET (2% *w*/*v*) (M_6_). A very simple process consisting of a conventional ES system was used for the preparation of the liposome based particles that involved a syringe pump adjusted with a flow of 1.0 mL/h, high voltage set at 16 kV, and a needle with an internal diameter of 0.2 mm. The collector used was a flat plate wrapped in aluminum foil fixed 25 cm away from the tip of the needle [32]. The temperature and humidity of the environment in which the ES was carried out were 25 ± 2 °C and 50 ± 5% RH. After obtaining the MPs, deionized (DI) water was added to generate the liposomes. The MPs without the drug (M_1_–M_5_) presented the same spherical morphology with some cutouts and with an average diameter of 0.9–1.0 μm. It was found that the PC concentration had little influence on the size of the MPs, but in contrast, the increase of the PC had an high influence on the agglomeration of the MPs [32]. The MPs loaded with the drug KET had the same characteristics as the rest of the MPs, but with increased polydispersity. Thus, in order to obtain liposomes, a total of 0.1 g of the MPs were used and added to 100 mL of DI water. The results from dynamic light scattering (DLS) showed consistent formation of large unilamellar vesicles (LUVs). At 25 °C, the formation of two distinct types of liposomes with sizes of 142 and 955 nm could be observed. When the temperature increased to 36 or 37 °C, only a single type of liposome was obtained, with a size of approximately 278 nm. The entrapment efficiency of KET in the liposomes from M_6_ was about 75.3% at 25 °C and 86.9% at 37 °C. Sustained drug release that could be regulated by the temperature was observed from these drug loaded materials [32]. At 25 °C, only 45% of the total drug loading was released after 110 hours, whereas at 37 °C, the drug release reached 90% over the same timeframe. Thus, these results demonstrated that this self-assembled liposome formed using ES could function as a great candidate as a drug delivery material [32]. Moreover, multilayer MPs can also be fabricated, which are represented in Figure 5h [53].

**Figure 6:**
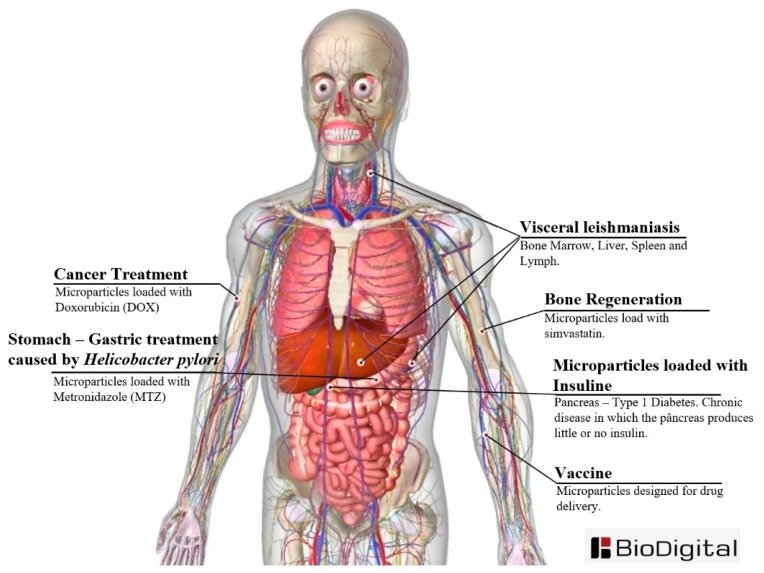
Examples of the MP applications in humans. Image generated by the BioDigital web application. Reproduced with permission. [65] Copyright 2019, BioDigital, Inc. Source: www.biodigital.com.

MPs have a wide range of applications and some of them are presented in the Supplemental Table S3 and illustrated in Figure 6. Some studies have focused on the investigation of the ability of MPs to encapsulate various drugs and its delivery system [36]. In this context, the preparation of hollow vesicle MPs through CES comprising of the polymer 2-methacryloyloxyethylphosphorylcholine (MPC) as the outer shell and calcium phosphate (CaP) and chitosan in the core have been disclosed by Matsuura and Maruyama [50]. Furthermore, various studies have focused on the investigation of a MP’s ability to encapsulate active agents and its delivery system. Suksamran et al. used BSA Albumin Fraction V (from bovine serum, 69,000 Da) for the production of MPs for encapsulation purposes [36]. The method allowed for the easy encapsulation of agents (such as dextran and bovine serum albumin (BSA)) with more than an 80% encapsulation efficiency. Moreover, Fei et al. demonstrated an air-controlled ES system for the production of graphene oxide (GO). The authors highlighted the success of the procedure despite no binders, organic solvents or extra conductive carbon [62]. Moreover, the obtained material demonstrated excellent cyclic stability and high specific capacity when tested in lithium (Li) half cells and fuel cells. Furthermore, other studies have also focused on designing MPs to be used as a vaccine [37] or for cancer [48], *Leishmania donovani* [34], gastritis caused by *Helicobacter pylori* (*H. pylori*) [55], and bone regeneration [30].

Suksamran et al. prepared calcium-alginate and calcium-yam-alginate MPs coated with chitosan (TM_65_CM_50_CS) by the ES technique in which ovalbumin (OVA) was used as an antigen model to induce immune responses in mice after oral vaccination [37]. The MPs were prepared by dissolving the alginate or alginate-yam and CaCl_2_ powder separately in water under magnetic stirring in order to obtain solutions of 1% (*w*/*v*) alginate and 4% (*w*/*v*) CaCl_2_. Subsequently, solutions containing various ratios between alginate and OVA (10%, 20%, and 40% *w*/*w*) were prepared. Next, the MPs were produced by placing the solutions (10 mL) in syringes and extruding them, using a needle with and internal needle diameter of 0.9 mm, into a solution containing 150 mL of CaCl_2_ solution (4% *w*/*v*). The voltage applied was 18 kV, the distance between the needle tip and collector was adjusted to 30 cm, and flow maintained at 1 mL/h [37]. The obtained OVA MPs loaded with calcium alginate (OVA-MP) and OVA MPs loaded on Ca-alginate-yam (OVA-YMP), were further coated with modified chitosan (TM_65_CM_50_CS) (CS-OVA-YMP and CS-OVA-MP). The MPs were obtained as a spherical shape with a diameter of 1.49 ± 0.11 (CS-OVA-MP) and 2.96 ± 0.23 μm (CS-OVA-YMP), respectively. Further cytotoxicity investigation confirmed the safety of the MPs and the overall study demonstrated the potential of the material as a vital candidate for the transport of vaccines and at the same time improving the immunogenicity of oral vaccines [37]. Additionally, MPs based on poly disulfide polyether urethane (PEU) as the core and PEG as the shell have been fabricated through CES [48]. Subsequent removal of the PEG provided a spherical morphology with an average diameter of 100 nm and could further successfully encapsulate the anticancer drug doxorubicin (DOX). The various parameters employed for the fabrication of the MPs were the following: using the solvent trifluoroethanol (TFE), polymer concentrations of the core (3%, 4%, and 5%) and the shell (10%), FR of 0.3 and 0.5 mL/h (core) and 1 mL/h (shell), a collector distance 17 cm, an applied voltage −11.06 kV, a temperature 25 ± 5 °C, and the RH was 30 ± 5% [48]. The designed MPs were aimed to be used in the treatment of Leishmaniasis affecting the bone marrow, liver, spleen, and lymph glands (Figure 6). In this context, according to the Centers for Disease Control (CDC), 90% of visceral leishmaniasis are untreated and result in mortality and therefore improved materials are badly needed to treat such diseases [34].

Furthermore, Duong et al. investigated for the first time the use of ES for the preparation of MPs for the treatment of a parasitic disease (leishmaniasis). The produced MPs comprised of Acetalated Dextran (Ac-DEX-MPs) or Ac-DEX-MPs with Sufentanil Tween (Ac-DEX-Tween-MPs) with encapsulated resiquimod (which is an imidazoquinoline employed to treat cutaneous leishmaniasis). The configurational parameters employed for the generation of the MPs were as follows: a stainless steel collector type with dimensions of 7.6 × 7.6 cm^2^, 908 μm external diameter needle, 603 μm internal diameter and a collector distance of 7 cm, the FR was 0.1 and 0.3 mL/h, and the applied voltage was −4.5 kV and EtOH was employed as the solvent [34]. Moreover, two surfactants (Tween 20 and 80) were added to improve the dispersion. Here, Tween 80 provided spherical MPs and easily suspended the MPs (Ac-DEX-Tween80-MPs) in a buffer, compared to when Tween 20 was employed. Encapsulation efficiency up to 60% (Ac-DEX-Tween80-MPs) was obtained with a release of 80% the first 0.5 h and the remaining amount after 8 h [34]. The in vivo performance of the MPs through intravenously injection into mice inoculated with *Leishmania*, showed a significant reduction in the bone marrow compared to controls (blank particles and phosphate buffer saline (PBS)) [34]. Moreover, MPs for the treatment of infections caused by the bacteria *H. pylori* have been prepared [40]. The stated bacteria reside in the stomach or intestine, and damage the protective barrier stimulating inflammation, which can cause symptoms such as abdominal pain and burning, as well as increasing the risk for the development of ulcers and cancer. In this study, ES deposition was successfully applied for the preparation of PLGA-based MPs. The synthesized material showed porous characteristics and the model drug selected was MTZ. MTZ has been widely used as one of the main components employed in therapies against *H. pylori*. The mean size of the MPs obtained ranged from 1.29 ± 0.01 to 8.39 ± 0.10 μm and with a cell viability >80% (through the MTT assay and with 400 μg/mL of MTZ loading) [40]. Other reports have also demonstrated the design of PLGA-based MPs as a drug delivery vehicle. Within this framework, Nath et al. devised PLGA based MPs by ES technology for the delivery of the drug simvastatin for bone regeneration MPs based on PEG-b-PLA encapsulated with DOX through subsequent rehydration after ES [30,66]. The results showed that this novel method provided a promising way to manufacture drug loaded polymer vesicles for intracellular administration of anticancer drugs.

It is noteworthy that particles in the sub-micro range (0.1–1.0 μm) have also been designed through ES such as macrovesicles and micro sized proteins for therapeutic and imaging applications [67,68,69]. Despite that one may think that there is not much difference between micrometer and submicrometer particles apart from the small size difference, they actually display fairly different properties such as surface area to volume ratios, differences in their fabrication, interactions with cells, and therefore provide different responses to the dynamic and complex in vivo environment making sub-micron and nanoparticles often more suitable for different applications. For instance, generally, microparticles are not expected to cross most biological barriers but on the other hand, the submicron counterparts display a higher likelihood of crossing cells and tissues [70,71].

## 3. Conclusions

Over the last several decades, great progress has been achieved in the development of ES technology for the design and fabrication of nano and MPs with various and tunable shapes and sizes for different applications. As has been presented, the ES technique is a relatively complex process in order to precisely control performance outcomes, since several factors have to be considered that can have a large impact on the final outcome. Parameters such as polymer concentration, solvent, applied voltage between needle and collector, distance between nozzle and collection plate, type of manifold, FR, type of syringe, ES chamber, needle gauge, as well as temperature and humidity all have a pivotal role in targeting the morphology and average size of the MPs generated. Therefore, a careful and strategic selection of important parameters suitable for the intended application, which eventually provides the desired particles needs to be thoughtfully investigated. In addition, there are parameters that determine whether the technique will form MPs or fibers (ESP). Here, with the ES technique, it was possible to observe that the adjustments of these such parameters can provide various MP properties including morphology such as spherical, elongated, or porous. Clearly, such changes in MP properties influence their biological activity and, thus, role in medicine. ES is a versatile technique that has shown great promise for drug encapsulation efficiency. Here, the employment of coaxial setting further offers excellent advantages, resulting in increasing encapsulation efficiency. Lastly, in the context of biomedical applications of these particles, the encapsulation of drugs for the treatment of cancer, visceral leishmaniasis, and bone regeneration among other applications show much promise for the use of ES.

## Figures and Tables

**Table 1:** Comparison of some common techniques to synthesize microparticles (MPs) via electrosprays (ES).

Method	Advantages	Disadvantages
Jet-milling, spray drying, micro grinding, suspension/emulsion polymerization, solvent evaporation, microfluidics	✓ Applies to versatile materials ✓ Carries a high range of organic or biological molecules ✓ Particles can be used in a controlled release system	❖ Requires multiple steps ❖ Encapsulation limitations (e.g., proteins or drugs) ❖ There are limitations to the use of high molecular weight polymers ❖ Use of various solvents ❖ Use of one or more surfactants ❖ Modification of the method for obtaining nanoparticles ❖ Waste generation
Electrospray (ES)	✓ Just one step ✓ High molecular weight polymers can be used ✓ Obtaining nanoparticles or sub-micrometer particles or microparticles ✓ Greater control on particle size ✓ Unique morphologies obtained ✓ Higher drug encapsulation efficiency ✓ In comparison with the other techniques, can encapsulate drugs without further attention to their degradation rate ✓ Low cost ✓ Easy and adaptable methodology ✓ Use of less solvents ✓ No use of surfactant (depending on the methodology) ✓ Low residue generation	❖ Obtaining few particles ❖ In some cases, a crosslinking agent is used

**Table 2:** Summary of the parameters that influence the ES process.

Sections	System Parameters	Content Description
Section 2.4.1.	Polymer concentration	Deals with the influence of variations in polymer concentration in the ES process
Section 2.4.2.	Type of solvent	Addresses the variation of solvent types and their mixtures in the ES process
Section 2.4.3.	Solution conductivity	Discusses the influence of solution conductivity on the ES process
Section 2.4.4.	Flow rate (FR)	Focuses on the discussion of the FR parameter and its influence on ES and the generated MPs morphology
Section 2.4.5.	Electric field	Discusses the influence of electric field on ES
Section 2.4.6.	Temperature and humidity	Adresses the influence of temperature and humidity on ES
Section 2.4.7.	Collector distance	Deals with the influence of collector distance
Section 2.4.8.	Types of collectors	Discusses the influence of collector type

## Supplementary Materials

The following are available online at https://www.mdpi.com/2079-4983/11/1/4/s1, Table S1: Summary, type of ES methodology and specification of parameters used, Table S2: Solvents and polymers used, and Table S3: Charges used in the microparticles and the treatment or objective.
