# Supplementary material for: Fabrication of Polymeric Microparticles by Electrospray: The Impact of Experimental Parameters
Source: J Funct Biomater. 2020 Jan 15;11(1):4. doi: 10.3390/jfb11010004 (PMC7151563; doi:10.3390/jfb11010004)
Supplement: Supplementary file 1 [file jfb-11-00004-s001.pdf]

Supplementary Material

Fabrication of Polymeric Microparticles by Electrospray: The Impact of Experimental Parameters

Alan Í. S. Morais [1], Ewerton G. Vieira [1], Samson Afewerki [2,3], Ricardo B. Sousa [4], Luzia M. C. Honorio [1], Anallyne N. C. O. Cambrussi [1], Jailson A. Santos [1], Roosevelt D. S. Bezerra [5], Josy A. O. Furtini [1], Edson C. Silva-Filho [1], Thomas J. Webster [6] and Anderson O. Lobo [1,*]

[1] LIMAV—Interdisciplinary Advanced Materials Laboratory, PPGCM—Materials Science and Engineering Graduate Program, UFPI—Federal University of Piaui, Teresina 64049-550, Brazil; alanicaro@gmail.com (A.Í.S.M.); ewertonvieira@ufpi.edu.br (E.G.V.); luzia_quimica@yahoo.com.br (L.M.C.H.); anallynecambrussi@gmail.com (A.N.C.O.C.); j.santospi@hotmail.com (J.A.S.); josy_osajima@yahoo.com.br (J.A.O.F.); edsonfilho@ufpi.edu.br (E.C.S.F.)
[2] Division of Engineering in Medicine, Department of Medicine, Harvard Medical School, Brigham & Women's Hospital, Cambridge, MA 02139, USA; samsom.afewerki@gmail.com
[3] Harvard-MIT Division of Health Science and Technology, Massachusetts Institute of Technology, MIT, Cambridge, MA 02139, USA
[4] Federal Institute of Education, Science and Technology of Tocantins, Dianápolis Campus, IFTO, Dianápolis 77300-000, Tocantins, Brazil; ricardo.sousa@ifto.edu.br
[5] Federal Institute of Education, Science and Technology of Piauí, Teresina-Central Campus, IFPI, Teresina 64000-040, Brazil; rooseveltdsb@ifpi.edu.br
[6] Department of Chemical Engineering, Northeastern University, Boston, MA 02115, USA; th.webster@northeastern.edu
* Correspondence: lobo@ufpi.edu.br; Tel.: +55 86 3237-1057

Table S1. Summary, type of electrospray methodology and specification of parameters used.

Methodology	System Parameters	Reference
EHDA	Chamber; D = 55 mm, 70 mm, 110 mm; ID = 0.39 mm; OD = 0.72 mm.	[1]
CES	Tubing: DI = 2mm e DE = 3mm; CFR = variation 0.20 a 1,00 mL/h; OD = 0.72 mm; SFR = variation de 4.0 e 5.0 mL/h; V = variation 8.0 a 15 kV.	[2]
ES	GS; V = 18 kV e 20 kV; D = 30 cm; CB = solution of $CaCl_2$; FR = 1 mL/h; NG = 20 (ID = 0,9 mm). Negative electrode attached to needle.	[3]
Multi-ES	The volume fraction of polymer varied with or without rhodamine; OD = 210 µm or 500 µm; M: stainless steel; OBS: Electrospray multiplexed, uses a micro-fabricated MES device consisting of a silicon nozzle chip and a puller.	[4]
ES with mobile mask.	FR = 0.5 mL/h; NG = 24; PS = 5 mL.	[5]
ES	The polymer concentration, the voltage, the collection distance and the feed rate were adjusted in different parameters; RH = 32%; T = 22 °C.	[6]
ES	Chamber; D = 15 cm; Teflon capillary; ID = 0,6 mm; FR = 5 mL/h; M – Metal; SP; Syringe = 2.5 mL; T = 20°C or 40°C; TC = Aluminum plate; V = 30 kV.	[7]
ES is assisted by floating electrodes.	D = 15 cm; FR – 0.5 mL/h; M: metallic; TC - interdigitated microelectrodes; V- Direct current (DC) 20 kV.	[8]

ES	C = 7 cm; FR – 0.1 e 0.3 mL/h; M: stainless steel; NG: 20 (908 μm OD, 603 μm ID); SP; T = stainless steel ground plate (7.6 x 7.6 cm^2); V = 4.5 kV.	[9]
ES	FR = 0.50 mL/h; ID = 0.55 mm; M: stainless steel; OD = 0.80 mm; PC = different proportions % w/V; SP; Silicone Tube; V = 15 kV;	[10]
ES	D = 30 cm; CB = Solution of $CaCl_2$; FR = 1 mL/h; GS; NG - 20 (ID 0,9 mm); V = 18 kV.	[11]
ES	Chamber; FR = variation 40 a 200 μL/h; M: stainless steel; OD - 210 μm ou 500 μm; The distance between the ring electrode (charged at 10 kV) and the collector was fixed at 12 cm with extractor, ring electrode and corona.	[12]
ES	D = 15 ou 25 cm; FR - 0,5 mL/h a 3 mL/h; GS 1 mL; MC: stainless steel; NG - 26 a 21 G; RH - 34% to 49%; SP; T - 23 to 24 °C; TC- standard aluminum sheets (20 × 20 cm^2) washed with 70% ethanol; V = 10 kV.	[13]
ES	D = 20 cm; FR – 0.2 e 4 $mL.h^{-1}$; steel needle with inner diameter of ID - 0.2, 0.5, 1 or 1.7 mm; PS; SP; V = 15 a 25 kV	[14]
CES	Coaxial needle; D = 10 cm; CB = double distilled water (dd-H_2O) or ammonium acetate buffer; CFR = 0.48 mL/h; CR; ID = 210 μm e OD = 600 μm; M: stainless steel; PS; SFR = 2.8 mL/h; V = 3 a 4 kV.	[15]
CES	V = 23 kV; Coaxial Needle; IN: ID = 330 e OD = 630 μm; CFR = 1.0; SFR = 0.5 ml/h; EN: ID = 1.0 e OD = 2.5 μm; D = 5.5 cm.	[16]
ES	D = 100 mm; FR = 900 μL/h; ID – 0.88 mm; OD = 1.27 mm; PC = variation 1% a 4% (w/v); SP; TC = aluminum foil; V=- + 15 kV; Perpendicular - horizontal	[17]
ES	D = 100 mm; FR = 600 to 1500 μL/h; SP; T = environment; TC = aluminum foil; V = +10 kV	[18]
ES	D = 25 cm; FR = 1.0 mL/h; ID = 0.25 mm; PS; RH = 50 ± 5%; 5 mL syringe; T = 25 ± 2 °C; TC = aluminum plate; V = 16 kV.	[19]
CES	Epoxy coated concentric spray head (EP); D = 20 cm.; CFR = 0.4, 0.7 e 1,0 mL/h; horizontal Two SP; RH = 52% ± 6%; SFR = 1.0 mL/h; T = 22 °C ± 3°C; TC = metal wrapped by aluminum foil; V = 20 kV.	[20]
ES	D = 10 cm; CB = aqueous solution of sodium tripolyphosphate (TPP) as a coagulant, 400 rpm stirring; FR = 5 mL/h; NG = 26 G; V = 23 kV.	[21]
ES ring	52 variations of conditions involving solvent, flow rate, collector distance and Needle Gauge (G); CR - located 6 mm above the tip of the needle; Syringe to Needle Solution Hose; RH = 40%; SP; Syringe de 5 mL; T = 25 °C.	[22]
ES	D = Distance nozzle and manifold - adjustable; ID = 0.4 mm; MC = stainless steel; Syringe de 5 ml; TC = aluminum foil; V = 5 kV; horizontal.	[23]
ES portátil	D = 6 cm; CB = $CaCl_2$ 0.102 M grounded; FR = 2 mL/h; PC = Alginate 2% (w/V); D = 20 cm; FR = 0.6 mL/h; PC = PLGA 2.5% (w/V); TC = Flat stainless steel catcher covered with aluminum foil.	[24]
ES	D = 15 cm; FR = 0.8 mL/h; SP; TC = standard aluminum sheets (15 × 15 cm^2) washed with 70% ethanol; V = 10 kV. (horizontal)	[25]
ES	D = 13 cm; CB = EtOH/PVA e EtOH, MeOH EtOH/OA, EtOH/DiW (50/50) EtOH/PS80, DiW (5, 22, 40°C); FR = 0.5 mL/h; NG - 23G (OD = 0.64 mm); V = 11 kV	[26]

ES and CES	5 mL glass syringe; 20G blunt point needle (The needle has the following dimensions: 51 mm length, 0.91 mm OD, and 0.60 mm ID); stainless steel (SS)collection plate; D = 10 cm; Collector - V = -2 kV; Needle - V = +10 and 11 kV; Outer Phase – FR = 0.85 mL/hr; Inner Phase - FR = 0.02 mL/hr;	[27]
CES	D = 17 cm; CFR = 0.3, 0.5 mL/h; PC = Nuclear layer (3%, 4%, 5%); RH = 30 ± 5 %, no rapid flow of air; SFR = 1 mL/h; T = 25 ± 5°C; V = 11.06 kV	[28]
CES with flow and air on the outer needle.	Coaxial Needle; Internal needle – NG -17 G; External needle - NG = 12 G; D = 20 cm; FR = 0.05 e 0.1 mL/min; Air pressure at 28 psi; SP; TC = Copper sheet; Substrate of porous carbon nanofibers.; Aluminum foil; V = 20 kV.	[29]
ES	syringe (1 mL); 1 mL h^{-1}; 20 kV; 0.62 mm inner diameter flat-ended metallic needle; D = 15 cm; RH 50% and 25°C	[30]
CES	D = 10 cm; PC = 4% w/v PLGA; FR = 12 μL/min; twin syringe pump; two high voltage DC power supplies; Positive voltage V = 10 kV, and negative voltage V = 8 kV. The TC = aluminum plate; The coaxial needle consists of an IN (ID = 0.26 mm; OD = 0.51 mm) and an ON (ID = 0.84 mm; OD = 1.27 mm);	[31]
CES	D = 5.0 cm; EN: ID e OD = 1.0 e 2.5 mm; IN: ID e OD = 330 e 630 μm, respectively; FR = 0.2 mL/h; SP; TC = Stainless steel dish containing disodium hydrogen phosphate solution; V = 23 kV.	[32]
CES	D = 5.0 cm; EN: ID e OD = 1.0 e 2.5 mm; IN: ID e OD = 330 e 630 μm, respectively; FR = 0.2 mL/h; SP; TC = Stainless steel dish containing disodium hydrogen phosphate solution; V = 23 kV.	[32]
CES	D = 5.0 cm; CFR e SFR = 0.2 mL/h; EN: ID = 1.0 mm; IN: ID = 140, 270 e 400 μm; TC = Stainless steel dish containing disodium hydrogen phosphate solution; V = 23 kV.	[33]
ES	D = 10 cm; FR = 0.7 e 1.4 mL/h; OD = 1.2 mm; PC = solutions of 5% by weight and 10% by weight of PVP/water; PS = plastic 20 mL inkjet syringe quarter-inch silicone tubing; SP; T = entre 21 e 24°C; TC = plate; V = 19, 22.7, 21 e 27 KV; WR = 1 cm diameter approximately 1 mm above the nozzle tip.	[34]
TES	Metallic ground ring electrode (diameter ~5 cm) located 5 cm below; MC – Stainless steel; EN: OD = 1.60 and ID = 1.07; IN: OD = 2.85 and ID = 2.26; CN: OD = 0.50 and ID = 0.31; Syringes connected to silicone tubes; SP = 3 units; V = 10 kV; FR = 10, 2.5 e 1.6 mL/h for the outer, middle and inner layers; PC = 10 % (w/v) de PCL	[35]
CES	D = 10 cm; GS; SFR = 0.5 mL/h; SP; V = +2.5 KV (needle); V = -8.0 KV (collector)	[36]
ES ring	liquid bath phase = 1 wt %, 2.5 wt %, 5 wt %, 7.5 wt %, or 10 wt % calcium chloride dihydrate; V = 5 kV; metallic ring; FR = 0,1 mL h^{-1}; borosilicate glass capillary (1 mm OD e 0,25 mm ID) at the tip coupled a micropipette puller com diameter of 50 μm (±5 μm)	[37]
ES	PC = 1 wt%, 3 wt%, 5 wt%, 10 wt%, 14 wt%, 15 wt%, 16 wt%, 17 wt%, and 18 wt%; MW = 14.8 kg/mol, 24.2 kg/mol, 52.7 kg/mol and 125.6 kg/mol; FR = 0.6 mL/h, 9 mL/h; V = 11 kV, 13 kV and 15 kV.	[38]
ES	PC = chitosan/PEG proportions (wt/wt) (90/10, 80/20, 70/30, 60/40); collector cylinder of 785 cm^2 covered by aluminum foil; drum rotation of 140 rpm, wash speed of 20 mm s^{-1};FR = 6 ml h^{-1}; scan speed of 40 mm s^{-1} under 25 kV voltage and 8 cm of needle-collector distance.	[39]

ES	V = 8 kV, nozzle: ID = 500 μm, D = 8 cm; CB= 2% (w/v) $CaCl_2$ was prepared in distilled water; header tank height = 20 cm	[40]
ES and CES	Eudragit solutions of 1.0% w/w and 2.0% w/w; 10 ml airtight syringes; needles of inner diameter 0.6 mm and 1.52 mm and outer diameter of 0.9 mm and 2.03 mm, respectively; Collector distance 250 mm; 6 μl min^{-1} and 12 μl min^{-1} for the inner and outer solution, respectively; 14 kV a 19 kV; room temperature 21 ± 1 °C and relative humidity of 40–60%	[41]
ES	PC= (2%–8% w/v); FR = (0.1–1 ml/hr); V = (10–20 kV); D = (5–15 cm); plastic syringe needle with a diameter of 0.8 mm; C =aluminum plate of 10 cm × 10 cm	[42]

Siglas: The electrohydrodynamic atomization = EHDA; Electrospray = ES; Electrospray Coaxial = CES; Electrospray tri-coaxial = TE; Polymer concentration = PC; Molecular weight = MW; Flow Rate = FR; core flow rate = CFR; shell flow rate = SFR; voltage = V; solvent type = ST; needle gauge = NG; collecting distance = D, collecting bath = CB; Internal diameter of the needle or capillary = ID; Outer diameter of needle or capillary = OD; External needle = EN, Internal needle = IN, Central needle = CN, Temperature = T, Relative humidity = RH; Distance from needle tip to ring = DNR, Wire Ring = WR; Copper ring = CR, Syringe pump = SP; Plastic syringe = PS; Glass syringe = GS; Type of collector = TC; Material collector = MC; Ethamol = EtOH; poly(vinyl alcohol) = PVA; Methanol = MeOH; Diestilated water = DiW; oleic acid = OA; polysorbat 80 = PS80;

Table 2. Solvents and polymers used.

Polymer	Solvent and surfactant	Reference
Poly lactide glycolide (PLGA), ethyl acetate copolymer (EVAC) and polycaprolactone (PCL) were used to make particles.	Dichloromethane (DCM) and acetonitrile (ACN) were used as organic solvent.	[1]
Poly(DL-lactide-co-glycolide) (PLGA 75:25, Mw = 90,000–126,000)	Acetonitrile	[2]
Sodium alginate powder (medium viscosity 20–4000 cps, low viscosity 250 cps)	Water	[3]
Poly(lactic-co-glycolic acid) (PLGA) (40 000–75 000 molecular weight, lactide/glycolide = 50:50, Sigma–Aldrich); pluronic F127 (Sigma–Aldrich)	dichloromethane (DCM) (Fisher Chemical, Waltham, MA); N,N-dimethylformamide (DMF) (Fisher Chemical, Waltham, MA)	[5]
PLGA (IV 0.55–0.75, Mw = 53.8 kDa, Lactel Absorbable Polymers, Durect Corporation)	2,2,2-trifluoroethanol (TFE, Reagent Plus grade, purity ⩾99%, Sigma–Aldrich)	[4]
Poly(methyl methacrylate) (PMMA, Mw = 110,000 g mol^{-1})	N,N-dimethylformamide (DMF)	[6]
Pluronic F127 (PL).	1-propanol	[7]
Polycaprolactone (PCL) (Sigma-Aldrich, Mn = 60 000)	Acetone	[8]
Poly(vinyl alcohol) (PVA); polymer acetalated dextran (Ac-DEX)	Dichloromethane (DCM)	[9]
Poly (D,L-lactic-co-glycolide) PLGA (85:15), Mw = 50,000–75,000) was also obtained from Sigma–Aldrich.	Methylene chloride or dichloromethane (DCM)	[10]
Low viscosity sodium alginate derived from brown algae (ALV, 250 cps) Chitosan ($TM_{65}CM_{50}CS$)	Water	[11]
Polycaprolactone (Mn = 84 kDa, PDI 1.53); Poly(ethylene glycol) Mn = 6 kDa and Mn = 35 kDa	Dichloromethane (DCM); Chloroform	[13]

polyvinyl alcohol (PVA)	Water	[15]
poly (β-aminoesters) (PBAE)	HCL; Acetate	[16]
Poly(vinylidene fluoride) (PVDF)	Tetrahydrofuran (THF); N,N-dimethyl formamide (DMF)	[14]
PNIPAAm (N-isopropylacrylamide (NIPAAm) + 2,2′-Azo bis(2-methylpropionitrile) (AIBN))	Chloroform; Ethanol	[19]
polyvinylpyrrolidone (PVP) K30 (molecular weight 58,000)	N, N-dimethylacetamide (DMAc); anhydrous ethanol	[20]
Poly(lactic-co-glycolic acid) (PLGA, molecular weight — 10 kDa)	Dichloromethane	[17]
Eudragit® RS PO (ERS, Mw = 32 kDa	Dichloromethane	[18]
Poly(lactic-co-glycolic) acid (PLGA) (50:50 weight ratio between the lactic and glycolic monomers)	2,2,2-Trifluoroethanol (TFE); dimethyl sulfoxide (DMSO); acetonitrile (ACN)	[12]
pCA-chitosan; pCA-HT-chitosan	Lactic acid	[21]
Poly(lactic-co-glycolic acid) (PLGA) copolymer (LA/GA: 50/50, Resomer RG503H, Mw = 36 KDa and Resomer RG502H, Mw = 12 KDa)	Dimethylformamide (DMF); tetrahydrofuran (THF)	[22]
Poly(lactic-co-glycolic acid) (PLGA 75:25, Mw 66-107 kD, Sigma-Aldrich); Alginate (sodium alginate, 100-300 cP, Sigma-Aldrich)	1,1,1,3,3,3- hexafluoroisopropanol (HFIP, Apollo Scientific Ltd., Cheshire, UK); deionised water	[24]
Poly(L-lactide) (PLA)	Chloroform; Surfactante triethyl benzyl ammonium chloride (TEBAC, $C_{13}H_{22}ClN$)	[23]
Poly(lactic-co-glycolic acid) (PLGA); Poly(ethylene glycol) (PEG) with Mn = 35 kDa	Chloroform, dichloromethane (DCM)	[25]
PLGA copolymer (LA/GA: 50/50, Resomer RG504H, Mw= 46 KDa) was purchased from Evonik Industries AG (Essen, Germany); Poly(vinyl alcohol) (PVA) Mw=130 KDa was bought from Sigma-Aldrich (USA)	Chloroform; methylene chloride; Acetonitrile; Dimethylformamide (DMF); Tetrahydrofuran (THF); Methanol; Ethanol; Polysorbat 80 (Tween 80)	[26]
Acetalated Dextran (Ace - DEX)	Ethanol/ethyl acetate/n-butanol; Polysorbat 80 (Tween 80)	[27]
poly disulfide polyether urethane (PEU) polyethylene glycol (PEG)	Trifluoroetanol (TFE, 99,9%)	[28]
Graphene oxide (GO)	In water suspension	[29]
Poly(ethylene glycol) monomethyl ether (mPEG, Mn = 2000 Da and 5000 Da), d,l-lactide (PDLA) and ε-caprolactone	Tetrahydrofuran (THF)	[30]
poly(lactic-co-glycolic) acid (PLGA) (MW = 10000–20000)	Acetonitrile	[31]
2-methacryloyloxyethyl phosphorylcholine (MPC) (MPC) - MPC polymer (Lipidure®-CM) and amine-modified MPC polymer (Lipidure®-NH01)	Triethylamine/acetate buffer (pH 8, 0.1 M); Ethanol	[32]
2-methacryloyloxyethyl phosphorylcholine (MPC) (MPC) - MPC polymer (Lipidure®-CM) and amine-modified MPC polymer (Lipidure®-NH01)	Triethylamine/acetate buffer (pH 8, 0.1 M); Ethanol	[33]
Polyvinylpyrrolidone (PVP)	Water	[34]
Poly(ε-caprolactone) (PCL) (Mw = 4.5 × 103 g/mol)	Glacial acetic acid (HAc)	[35]
Acetalated Dextran (Ace-DEX)	Ethyl acetate:n-butanol:ethanol	[36]

Alginic acid sodium salt (Sigma–Aldrich Corp., St. Louis, MO, USA)	Deionized (DI) water	[37]
chitosan (MW: 120.106 g mol^{-1}; degree of deacetylation 85%), PEG (MW 20.000 g mol^{-1}) and hyaluronic acid 800.000 g mol^{-1}	2% acetic acid in double distilled water (v/v)	[39]
Alginate (MW ~ 120 k Da, guluronic to mannuronic ratio of 1.7:1) (polissacarideo); chitosan [viscosity average molecular weight MWη ~ 50–190 kDa, degree of deacetylation (DD): 75–85%] and chitosan (MWη ~ 310–375 kDa, DD < 75%)	Tistilled water, acetic acid	[40]
Eudragit L100-55 (MW = 320 000 g mol^{-1})	Isopropanol	[41]
Wheat gluten	Acetic acid, ethanol	[42]

Table 3. Charges used in the microparticles and the treatment or objective.

Charge	Treatment / objective	Reference
Bovine serum albumin (BSA) and lysozyme	Encapsulate bovine serum albumin (BSA) and lysozyme	[2]
BSA (Albumin Fraction V from bovine serum, 69 000 Da)	Charging potential	[3]
Insulin encapsulation	Selected as a peptide template	[7]
Encapsulation of resiquimod	Treatment of *Leishmania donovani*	[9]
Encapsulation of 90% of simvastatin	Drug release and application in bone tissue engineering	[10]
Albumin from chicken egg white (ovalbumin [OVA], Grade V, MW = 44 kDa)	Antigen model to induce immune responses in mice after oral vaccination	[11]
Metronidazole (MTZ)	Gastric treatment caused by *Helicobacter pylori*	[18]
Metronidazole (MTZ)	Gastric treatment caused by Helicobacter pylori	[17]
Ketoprofen (KET)	Drug Release	[19]
Acyclovir (ACY)	Low water soluble model drug	[20]
Piperine (PIP)	Reduce gastrointestinal irritation (microparticle loaded with PIP)	[21]
Rifampin	Model drug	[23]
Recombinant vascular endothelial growth factor (VEGF), Recombinant human bone morphogenetic protein-7 (BMP-7)	Bone tissue	[25]
Recombinant protective antigen (rPA)	*Bacillus Anthracis*	[27]
Loaded with doxorubicin (DOX)	Efficiency and release	[28]
Graphene oxide (GO)	Battery electrodes	[29]
Doxorubicin (DOX)	An anticancer drug used to demonstrate the approach to the application of encapsulation and delivery of hydrophilic drug	[30]
Artemether	Produce artemether-loaded PLGA-MPs for enhanced bioavailability in malarial therapy.	[31]
Yeast Saccharomyces cerevisiae Kyokai No. 7	Encapsulation and culture of living cells in vesicles	[33]
Dextran and BSA encapsulation	Release study	[32]

Murabutide, ovalbumina (OVA)	As an adjuvant model or antigen, respectively.	[36]
They used 0.945 μm diameter Sera-Mag carboxylate-modified magnetic speed-beads. BT-474 breast cancer cells; BT-474 breast cancer cells	For generation and magnetic microparticles and another for cellular encapsulation model	[37]
Caffeine	Hydrophilic model compound to evaluate the efficiency of microparticle encapsulation	[40]
Prednisolone	Inflammatory bowel disease (IBD) (ulcerative colitis and Crohn's disease)	[41]

References

1. Yao, J.; Kuang Lim, L.; Xie, J.; Hua, J.; Wang, C.-H. Characterization of electrospraying process for polymeric particle fabrication. *J. Aerosol Sci.* **2008**, *39*, 987–1002.
2. Xie, J.; Ng, W.J.; Lee, L.Y.; Wang, C.H. Encapsulation of protein drugs in biodegradable microparticles by co-axial electrospray. *J. Colloid Interface Sci.* **2008**, *317*, 469–476.
3. Suksamran, T.; Opanasopit, P.; Rojanarata, T.; Ngawhirunpat, T.; Ruktanonchai, U.; Supaphol, P. Biodegradable alginate microparticles developed by electrohydrodynamic spraying techniques for oral delivery of protein. *J. Microencapsul.* **2009**, *26*, 563–570.
4. Almería, B.; Deng, W.; Fahmy, T.M.; Gomez, A. Controlling the morphology of electrospray-generated PLGA microparticles for drug delivery. *J. Colloid Interface Sci.* **2010**, *343*, 125–133.
5. Li, X.; MacEwan, M.R.; Xie, J.; Siewe, D.; Yuan, X.; Xia, Y. Fabrication of Density Gradients of Biodegradable Polymer Microparticles and Their Use in Guiding Neurite Outgrowth. *Adv. Funct. Mater.* **2010**, *20*, 1632–1637.
6. Wang, H.; Liu, Q.; Yang, Q.; Li, Y.; Wang, W.; Sun, L.; Zhang, C.; Li, Y. Electrospun poly(methyl methacrylate) nanofibers and microparticles. *J. Mater. Sci.* **2010**, *45*, 1032–1038.
7. Bussano, R.; Chirio, D.; Costa, L.; Turci, F.; Trotta, M. Preparation and Characterization of Insulin-Loaded Lipid-Based Microspheres Generated by Electrospray. *J. Dispers. Sci. Technol.* **2011**, *32*, 1524–1530.
8. Zhang, X.; Zhao, Y. Programmable patterning of polymeric microparticles by floating electrodes-assisted electrospray. *J. Micromechanics Microengineering* **2012**, *22*, 047001.
9. Duong, A.D.; Sharma, S.; Peine, K.J.; Gupta, G.; Satoskar, A.R.; Bachelder, E.M.; Wyslouzil, B.E.; Ainslie, K.M. Electrospray Encapsulation of Toll-Like Receptor Agonist Resiquimod in Polymer Microparticles for the Treatment of Visceral Leishmaniasis. *Mol. Pharm.* **2013**, *10*, 1045–1055.
10. Nath, S.D.; Son, S.; Sadiasa, A.; Min, Y.K.; Lee, B.T. Preparation and characterization of PLGA microspheres by the electrospraying method for delivering simvastatin for bone regeneration. *Int. J. Pharm.* **2013**, *443*, 87–94.
11. Suksamran, T.; Ngawhirunpat, T.; Rojanarata, T.; Sajomsang, W.; Pitaksuteepong, T.; Opanasopit, P. Methylated N-(4-N,N-dimethylaminocinnamyl) chitosan-coated electrospray OVA-loaded microparticles for oral vaccination. *Int. J. Pharm.* **2013**, *448*, 19–27.
12. Almería, B.; Gomez, A. Electrospray synthesis of monodisperse polymer particles in a broad (60nm–2μm) diameter range: guiding principles and formulation recipes. *J. Colloid Interface Sci.* **2014**, *417*, 121–130.
13. Bock, N.; Dargaville, T.R.; Woodruff, M.A. Controlling microencapsulation and release of micronized proteins using poly(ethylene glycol) and electrospraying. *Eur. J. Pharm. Biopharm.* **2014**, *87*, 366–377.
14. Correia, D.M.; Gonçalves, R.; Ribeiro, C.; Sencadas, V.; Botelho, G.; Ribelles, J.L.G.; Lanceros-Méndez, S. Electrosprayed poly(vinylidene fluoride) microparticles for tissue engineering applications. *RSC Adv.* **2014**, *4*, 33013–33021.
15. Duong, A.D.; Ruan, G.; Mahajan, K.; Winter, J.O.; Wyslouzil, B.E. Scalable, Semicontinuous Production of Micelles Encapsulating Nanoparticles via Electrospray. *Langmuir* **2014**, *30*, 3939–3948.

16. Funasaki, Y.; Tsuchiya, E.; Maruyama, T. Hollow giant lipid vesicles prepared by coaxially electrospraying solutions of phospholipid and degradable polyelectrolyte. *Colloid Polym. Sci.* **2014**, *292*, 3049–3053.
17. Hao, S.; Wang, Y.; Wang, B.; Deng, J.; Zhu, L.; Cao, Y. Formulation of porous poly(lactic-co-glycolic acid) microparticles by electrospray deposition method for controlled drug release. *Mater. Sci. Eng. C* **2014**, *39*, 113–119.
18. Hao, S.; Wang, Y.; Wang, B.; Zou, Q.; Zeng, H.; Chen, X.; Liu, X.; Liu, J.; Yu, S. A novel gastroretentive porous microparticle for anti-Helicobacter pylori therapy: Preparation, in vitro and in vivo evaluation. *Int. J. Pharm.* **2014**, *463*, 10–21.
19. Jin, C.; Li, H.; Williams, G.R.; Wei, R.; Nie, H.; Quan, J.; Zhu, L. Self-assembled liposomes from electrosprayed polymer-based microparticles. *Colloid Polym. Sci.* **2014**, *292*, 2325–2334.
20. Liu, Z.-P.; Cui, L.; Yu, D.-G.; Zhao, Z.-X.; Chen, L. Electrosprayed core–shell solid dispersions of acyclovir fabricated using an epoxy- coated concentric spray head. *Int. J. Nanomedicine* **2014**, *9*, 1967.
21. Pengpong, T.; Sangvanich, P.; Sirilertmukul, K.; Muangsin, N. Design, synthesis and in vitro evaluation of mucoadhesive p-coumarate-thiolated-chitosan as a hydrophobic drug carriers. *Eur. J. Pharm. Biopharm.* **2014**, *86*, 487–497.
22. Jafari-Nodoushan, M.; Barzin, J.; Mobedi, H. Size and morphology controlling of PLGA microparticles produced by electro hydrodynamic atomization. *Polym. Adv. Technol.* **2015**, *26*, 502–513.
23. Lu, J.; Hou, R.; Yang, Z.; Tang, Z. Development and characterization of drug-loaded biodegradable PLA microcarriers prepared by the electrospraying technique. *Int. J. Mol. Med.* **2015**, *36*, 249–254.
24. Mouthuy, P.-A.; Groszkowski, L.; Ye, H. Performances of a portable electrospinning apparatus. *Biotechnol. Lett.* **2015**, *37*, 1107–1116.
25. Bock, N.; Dargaville, T.R.; Kirby, G.T.S.; Hutmacher, D.W.; Woodruff, M.A. Growth Factor-Loaded Microparticles for Tissue Engineering: The Discrepancies of In Vitro Characterization Assays. *Tissue Eng. Part C Methods* **2016**, *22*, 142–154.
26. Faramarzi, A.R.; Barzin, J.; Mobedi, H. Effect of solution and apparatus parameters on the morphology and size of electrosprayed PLGA microparticles. *Fibers Polym.* **2016**, *17*, 1806–1819.
27. Gallovic, M.D.; Schully, K.L.; Bell, M.G.; Elberson, M.A.; Palmer, J.R.; Darko, C.A.; Bachelder, E.M.; Wyslouzil, B.E.; Keane-Myers, A.M.; Ainslie, K.M. Acetalated Dextran Microparticulate Vaccine Formulated via Coaxial Electrospray Preserves Toxin Neutralization and Enhances Murine Survival Following Inhalational Bacillus Anthracis Exposure. *Adv. Healthc. Mater.* **2016**, *5*, 2617–2627.
28. Dai, H.-F.; Chen, J.-M.; Wang, L.-Q. Preparation of stimuli responsive nanoparticles using coaxial electrospray template removal method and its application as drug delivery system. *Acta Polym. Sin.* **2017**, 1947–1954.
29. Fei, L.; Yoo, S.H.; Villamayor, R.A.R.; Williams, B.P.; Gong, S.Y.; Park, S.; Shin, K.; Joo, Y.L. Graphene Oxide Involved Air-Controlled Electrospray for Uniform, Fast, Instantly Dry, and Binder-Free Electrode Fabrication. *ACS Appl. Mater. Interfaces* **2017**, *9*, 9738–9746.
30. Li, W.; Liu, S.; Yao, H.; Liao, G.; Si, Z.; Gong, X.; Ren, L.; Wang, L. Microparticle templating as a route to nanoscale polymer vesicles with controlled size distribution for anticancer drug delivery. *J. Colloid Interface Sci.* **2017**, *508*, 145–153.
31. Mangrio, F.A.; Dwivedi, P.; Han, S.; Zhao, G.; Gao, D.; Si, T.; Xu, R.X. Characteristics of Artemether-Loaded Poly(lactic- co -glycolic) Acid Microparticles Fabricated by Coaxial Electrospray: Validation of Enhanced Encapsulation Efficiency and Bioavailability. *Mol. Pharm.* **2017**, *14*, 4725–4733.
32. Matsuura, T.; Maruyama, T. Calcium phosphate-polymer hybrid microparticles having functionalized surfaces prepared by a coaxially electrospray technique. *Colloids Surfaces A Physicochem. Eng. Asp.* **2017**, *526*, 64–69.
33. Matsuura, T.; Maruyama, T. Hollow phosphorylcholine polymer vesicles prepared by a coaxial electrospray technique. *Colloid Polym. Sci.* **2017**, *295*, 1251–1256.
34. Sander, S.; Tischler, C.; Achelis, L.; Henein, H.; Fritsching, U. Production of polymer microparticles by electrospray atomization. *At. Sprays* **2017**, *27*, 457–464.

35. Zhang, C.; Yao, Z.-C.; Ding, Q.; Choi, J.J.; Ahmad, Z.; Chang, M.-W.; Li, J.-S. Tri-Needle Coaxial Electrospray Engineering of Magnetic Polymer Yolk–Shell Particles Possessing Dual-Imaging Modality, Multiagent Compartments, and Trigger Release Potential. *ACS Appl. Mater. Interfaces* **2017**, *9*, 21485–21495.
36. Chen, N.; Johnson, M.M.; Collier, M.A.; Gallovic, M.D.; Bachelder, E.M.; Ainslie, K.M. Tunable degradation of acetalated dextran microparticles enables controlled vaccine adjuvant and antigen delivery to modulate adaptive immune responses. *J. Control. Release* **2018**, *273*, 147–159.
37. Jeyhani, M.; Mak, S.Y.; Sammut, S.; Shum, H.C.; Hwang, D.K.; Tsai, S.S.H. Controlled Electrospray Generation of Nonspherical Alginate Microparticles. *ChemPhysChem* **2018**, *19*, 2113–2118.
38. Lee, H.; An, S.; Kim, S.; Jeon, B.; Kim, M.; Kim, I.S. Readily Functionalizable and Stabilizable Polymeric Particles with Controlled Size and Morphology by Electrospray. *Sci. Rep.* **2018**, *8*, 1–10.
39. Mutlu, E.C.; Ficai, A.; Ficai, D.; Yildirim, A.B.; Yildirim, M.; Oktar, F.N.; Demir, A. Chitosan/poly(ethylene glycol)/hyaluronic acid biocompatible patches obtained by electrospraying. *Biomed. Mater.* **2018**, *13*, 055011.
40. Nikoo, A.M.; Kadkhodaee, R.; Ghorani, B.; Razzaq, H.; Tucker, N. Electrospray-assisted encapsulation of caffeine in alginate microhydrogels. *Int. J. Biol. Macromol.* **2018**, *116*, 208–216.
41. Shams, T.; Illangakoon, U.E.; Parhizkar, M.; Harker, A.H.; Edirisinghe, S.; Orlu, M.; Edirisinghe, M. Electrosprayed microparticles for intestinal delivery of prednisolone. *J. R. Soc. Interface* **2018**, *15*.
42. Tapia-Hernández, J.A.; Rodríguez-Félix, D.E.; Plascencia-Jatomea, M.; Rascón-Chu, A.; López-Ahumada, G.A.; Ruiz-Cruz, S.; Barreras-Urbina, C.G.; Rodríguez-Félix, F. Porous wheat gluten microparticles obtained by electrospray: Preparation and characterization. *Adv. Polym. Technol.* **2018**, *37*, 2314–2324.
